# Calibrating Snakehead Diversity with DNA Barcodes: Expanding Taxonomic Coverage to Enable Identification of Potential and Established Invasive Species

**DOI:** 10.1371/journal.pone.0099546

**Published:** 2014-06-10

**Authors:** Natasha R. Serrao, Dirk Steinke, Robert H. Hanner

**Affiliations:** 1 Biodiversity Institute of Ontario, University of Guelph, Guelph, Ontario, Canada; 2 Department of Integrative Biology, University of Guelph, Guelph, Ontario, Canada; University of Idaho, United States of America

## Abstract

Detecting and documenting the occurrence of invasive species outside their native range requires tools to support their identification. This can be challenging for taxa with diverse life stages and/or problematic or unresolved morphological taxonomies. DNA barcoding provides a potent method for identifying invasive species, as it allows for species identification at all life stages, including fragmentary remains. It also provides an efficient interim taxonomic framework for quantifying cryptic genetic diversity by parsing barcode sequences into discontinuous haplogroup clusters (typical of reproductively isolated species) and labelling them with unique alphanumeric identifiers. Snakehead fishes are a diverse group of opportunistic predators endemic to Asia and Africa that may potentially pose significant threats as aquatic invasive species. At least three snakehead species (*Channa argus*, *C. maculata*, and *C. marulius*) are thought to have entered North America through the aquarium and live-food fish markets, and have established populations, yet their origins remain unclear. The objectives of this study were to assemble a library of DNA barcode sequences derived from expert identified reference specimens in order to determine the identity and aid invasion pathway analysis of the non-indigenous species found in North America using DNA barcodes. Sequences were obtained from 121 tissue samples representing 25 species and combined with public records from GenBank for a total of 36 putative species, which then partitioned into 49 discrete haplogroups. Multiple divergent clusters were observed within *C. gachua*, *C. marulius, C. punctata and C. striata* suggesting the potential presence of cryptic species diversity within these lineages. Our findings demonstrate that DNA barcoding is a valuable tool for species identification in challenging and under-studied taxonomic groups such as snakeheads, and provides a useful framework for inferring invasion pathway analysis.

## Introduction

Invasive species are a leading cause of decline and extinction of native fishes globally [1]. One group of potential aquatic invaders are snakehead fishes (family Channidae), which have generated concern due to their wide-ranging diet, parental care, and successful establishment [2]. Snakehead species inhabit freshwater ecosystems and are divided into two geographically isolated genera. The genus *Parachanna* consists of three nominal species that are native to Africa, whereas *Channa* is represented by at least 30 species native to Asia [3] (Table 1). Three snakehead species have established populations in the United States due to human-mediated introductions [2]: the blotched snakehead, (*C. maculata*) became established in Hawaii prior to 1900, while the bullseye snakehead (*C. marulius*) has been established in Florida since 2000 [4]. Although formal risk assessment has identified habitat suitability for both species in the southern United States and parts of Mexico [5], there is very little information available on their potential invasiveness and/or ecological effects. The northern snakehead (*C. argus*) is of far more concern as a potential North American invader. Within the last decade, the northern snakehead (*C. argus*) has established multiple populations in the eastern United States [4]. Their rapid colonization and spread, as well as the species' tolerance for colder temperatures have identified *C. argus* as being of significant concern [2], [5], [6]. Correctly identifying snakehead species is therefore necessary, as they vary in their ecological requirements and potential invasive ability [5], [7].

**Table 1:** List of Channidae species, their distributions and type localities as accepted by the Catalogue of Fishes [3].

Channidae Species	Reference	Distribution	Type Locality	Barcode coverage?
*C. amphibeus*	McClelland 1845	Southern Asia: native to northeastern India and Bhutan	Vicinity of tributaries of Teesta River	No
*C. argus*	Cantor 1842	Native range: China, Korea and Russia; introduced elsewhere, including the Maryland, U.S.A.	Zoushan Dao, China	Yes
*C. asiatica*	Linnaeus 1758	China; introduced elsewhere	Asia	Yes
*C. aurantimaculata*	Musikasinthorn 2000	Northern Assam, India	Dibrugarh town, Digrugarh, Assam, India	Yes
*C. bankanensis*	Bleeker 1853	Indonesia and Malaysia	Bangka Island, Malaysia	Yes
*C. baramensis*	Steindachner 1901	Malaysia	Baram River, northern Sarawak	No
*C. barca*	Hamilton 1822	India, Nepal, Bhutan and Bangladesh	Brahmaputra River, near Goalpara, Assam, India	Yes
*C. bleheri*	Vierke 1991	Brahmaputra basin, India	Upper part of Dibru River, near Guijan, Brahmaputra Aiver basin, northern Assam, India	Yes
*C. burmanica*	Chaudhuri 1919	Myanmar	Putao Plains	Yes
*C. cyanospilos*	Bleeker 1853	Indonesia and Malaysia	Bandar Lampung, southern Sumatra, Indonesia	No
*C. diplogramma*	Day 1865	Southern India	N/A	Yes
*C. gachua*	Hamilton 1822	Southern and southeastern Asia: Afganistan and Iran to China and Malaysia and Indonesia.	Ponds and ditches of Bengal, India	Yes
*C. harcourtbutleri*	Annandale 1918	Myanmar	Southern Shan State, Myanmar	No
*C. lucius*	Cuvier 1831	Southeastern Asia	Java	Yes
*C. maculata*	Lacepède 1801	Asia; native range southern China and northern Vietnam; introduced elsewhere	N/A	Yes
*C. marulioides*	Bleeker 1851	Indonesia and Malaysia	Sambas, Kalimantan, Indonesia	No
*C. marulius*	Hamilton 1822	Southern and southeastern Asia: Pakistan to southern China, Thailand, Laos and Vietnam; introduced elsewhere, including southern Florida	Gangetic provinces, India	Yes
*C. melanoptera*	Bleeker 1855	Indonesia	Kapuas River, Pontianak	No
*C. melanostigma*	Geetakumari&Vishwanath 2011	Lohit River, Brahmaputra River drainage, India	N/A	No
*C. melasoma*	Bleeker 1851	Native range: Malaysia and Indonesia; introduced elsewhere	Sambas, western Borneo	Yes
*C. micropeltes*	Cuvier 1831	Southeastern Asia	Java, Indonesia	Yes
*Channa nox*	Zhang, Musikasinthorn& Watanabe 2002	Only from near Hepu, Guangxi, China	Nanliu River basin, vicinity of Hepu, Guangxi Province, China	No
*C. orientalis*	Bloch & Schneider 1801	Southern and southeastern Asia	Habitat in Indian orientale (east India)	Yes
*C. ornatipinnis*	Britz 2008	WalounChaung, Myanmar	N/A	Yes
*C. panaw*	Musikasinthorn 1998	Myanmar	Yangon fish market, Yangon Myanmar	Yes
*C. pleurophthalma*	Bleeker 1851	Indonesia	Bandjarmasin, Borneo, Indonesia	Yes
*C. pulchra*	Britz 2007	KyeinthaliChaung, Myanmar	N/A	Yes
*C. punctata*	Bloch 1793	Southern Asia: Afganistan, Pakistan, India, Sri Lanka, Bangladesh, Myanmar, Thailand, Malaysia and China; introduced elsewhere	Rivers and lakes of Malabar coast, southwestern India	Yes
*C. stewartii*	mnPlayfair 1867	India, Bangladesh, and Nepal, possible further east	Cachar, Assam, India	Yes
*C. striata*	Bloch 1793	Southern Asia: Native range Pakistan to China, Thailand, Malayasia and Indonesia; introduced elsewhere	Malabar, southwestern India	Yes
*P. africana*	Steindachner 1879	West-central Africa: Ghana, Benin and Nigeria	Lagos, Nigeria	Yes
*P. insignis*	Sauvage 1884	West-central Africa: Gabon and Democratic Republic of Congo	Upper Ogooue River, Gabon	Yes
*P. obscura*	Günther 1861	Widepread in western and central Africa	West Africa	Yes

Despite the attention snakeheads have received, there are substantial difficulties for accurate species identification [8]. Existing taxonomic keys are limited to local geographic regions, and there is no comprehensive morphological key for the Channidae. At present, the most comprehensive listing of snakehead species is that of Courtenay and Williams [2], which provides summary species accounts but no keys for their identification. Moreover, several “species” currently circumscribed are thought to represent species complexes. While this issue has received recent attention with a number of new species descriptions [9]–[11], a clear picture of snakehead diversity remains elusive.

Genetic calibration of snakehead diversity and interspecies differences could significantly aid taxonomic resolution within the group. Orell and Weight [12] identified seven distinct and locally restricted mitochondrial (mtDNA) control region haplotypes among established populations of *C. argus* in eastern North American waters, suggesting multiple independent introductions from different maternal sources. This was supported by King and Johnson [13], who similarly concluded that there were multiple introductions, based on microsatellite analyses. Lakra et al. [14] sequenced 16S and cytochrome *c* oxidase subunit I (COI) mtDNA of eight Indian snakehead species, while Bhat et al. [15] used Random Amplified Polymorphic DNA (RAPDs) in seven of the same species; both studies aimed to test the utility of the respective approaches to discriminate species. The most comprehensive coverage of snakehead diversity was assessed in a phylogenetic study by Li et al. [16], which sequenced individuals from 20 species and focused primarily on the NADH 1 and 2 mitochondrial genes (ND-1 and ND-2). Despite the substantial molecular studies on snakehead fishes, the lack of directly comparable sequence data across species between studies, limits the utility of this body of data from the literature for resolving taxonomic boundaries or identifying non-natives using a molecular approach.

As a tool for species identification and discovery, DNA barcoding uses a standardized ∼650 base-pair segment of the mitochondrial 5′ COI gene region to map animal diversity and identify cryptic species [17]. The Fish Barcode of Life (FISH-BOL; [18]) campaign was launched to create a barcode reference sequence library for all fishes in order to facilitate their identification at all life stages and to expand knowledge of their geographic distributions and varied life histories. Barcoding has been successfully applied to both freshwater and marine fishes on continental scales e.g. [19]–[21]. It can enhance the accuracy of species identifications e.g. [22], [23] and aid in cryptic species detection [24]. Barcode records for snakeheads are beginning to appear in the literature due to a number of regionally [14], [25], [26] and taxonomically [27] focused efforts, but many species have yet to be characterized. The major objectives of this study were to extend the library of DNA barcode sequences derived from expert-identified reference specimens and to assess the utility of barcoding for elucidating the identity of non-native snakeheads and their entry pathways.

## Materials and Methods

### Ethics Statement

No specific permits were needed for this study. Museum collections and other laboratories donated specimens used for this study. Permission from the relevant museums/institutions to access the collections were obtained from: the California Academy of Sciences, Florida Museum of Natural History, New York State Department of Environmental Conservation Bureau of Fisheries, Ministry of Natural Resources, North Carolina Museum of Natural Sciences, Cornell University, Queensland University of Technology, Royal Ontario Museum, Simon Fraser University, The Academy of Natural Sciences of Drexel University, University of British Columbia, University of Copenhagen, University of Florida, University of Kerala, Universiti Sains Malaysia, and the Virginia Department of Game and Inland Fisheries.

A total of 121 snakehead specimens were sequenced for the mitochondrial 5′ COI barcoding region. Specimens were sourced from various institutions worldwide, including expert-identified reference specimens derived from within their native ranges, as well as those obtained from outside the known range of snakeheads (e.g. established invaders, from the aquarium trade, or from food markets). Voucher specimen information and digital images (where applicable) were deposited in the Barcode Of Life Database (BOLD website. Available: http://www.boldsystems.org. Accessed 2014 May 21. [28]) following recommendations of the FISH-BOL collaborators protocol [29]. Vouchers were retained for all but two specimens (NRSC040-11 and NRSC042-11). A “reference” sequence in this study is defined as one that was obtained from a native range or a sequence that was imported from GenBank (GenBank website. Available: www.ncbi.nlm.nih.gov. Accessed 2014 May 21). All pertinent specimen information is accessible through the BOLD project DSCHA ‘Family Channidae’ (BOLD website. Available: http://www.boldsystems.org. Accessed 2014 May 21) or the DOI for data set: (DOI website. Available: dx.doi.org/10.5883/DS-DSCHA. Accessed 2014 May 21).

DNA was extracted using a Qiagen DNeasy Blood & Tissue Kit (QIAGEN) following the manufacturer's instructions with some exceptions: after adding AW2, spin columns were dried through a final centrifugation at 17,000×*g* for 5 minutes; sample DNA was eluted with 50 µL of AE buffer and centrifuged at 6,000×*g* for 1 minute, and the same 50 µL of AE buffer was then re-eluted with a final centrifugation at 6,000×*g* for 1 minute in order to increase the DNA concentration. Each 12.5 µL PCR reaction consisted of 2 µL of template DNA, 6.25 µL 10% trehalose, 2 µL ddH_2_O, 0.625 µL MgCl_2_ [50 mM], 0.0625 µL dNTPs [10 mM], 0.06 µL Platinum *Taq* (Invitrogen), 0.10 µL [0.01 mM] each of the universal fish COI cocktail primers C_FishF1t1 and C_FishR1t1 [30] and 1.25 µL 10X PCR buffer (Invitrogen). PCR thermocycling conditions were an initial hot start of 94°C for 2 min, 25 cycles of [denaturation at 94°C for 30 s, annealing at 52°C for 40 s and extension at 72°C for 1 min], with a final extension at 72°C for 10 min. PCR products were visualised using 2% agarose gel E-Gel96 Pre-cast Agarose Electrophoresis System (Invitrogen). Only amplicons with single, intense bands were sequenced.

Each sequencing reaction consisted of 1 µL of PCR product along with 1 µL BIG DYE 3.1 reagent (Applied Biosystems, Inc), 1 µL M13F/M13R primer [31], 10 µL ddH_2_O and 1 µL 5X sequencing buffer (Invitrogen). The thermocycling profile was an initial hot start 96°C for 2 min, followed by 30 cycles of [denaturation at 96°C for 30 s, annealing at 55°C for 15 s, and an extension at 60°C for 4 min]. PCR products were bidirectionally sequenced and run on an ABI 3730 capillary sequencer (Applied Biosystems). Sequencher 4.05 (GeneCodes) was used to trim primers, assemble and manually edit bidirectional contigs from raw electropherogram “trace” files.

Sequence contigs (and their supporting trace files) were uploaded to BOLD [28], and combined with other published sequences from GenBank [32]. Sequences were aligned using a Hidden Markov Model alignment of translated COI amino acid sequences [28]. Aligned sequences were used to generate pairwise or p-distances [33] to infer a neighbour-joining phenogram of sequence divergences using MEGA 5 [34] to provide a visual depiction of the barcode variation among and between species, with bootstrap analysis (based on 500 replications). Sequence data were also parsed into molecular operational taxonomic units (MOTUs) using the RESL (Refined Single Linkage Analysis) algorithm and subsequently annotated with Barcode Index Numbers (BINs), as implemented on version 3 of BOLD [28]. This approach combines single linkage clustering and Markov clustering to recognize gaps in sequence space that correlate with species boundaries by optimizing MOTU partitions using the Silhouette index and uniquely labelling each MOTU with a Barcode Index Number (detailed in [35]). Concordance was assessed between BINs and specimens that were morphologically identified to species by characterizing the discordance (or lack thereof) between morphological species identifications and BIN clusters into one of four categories (MATCH, SPLIT, MERGE or MIXTURE). When members of a single species clustered within a single discrete BIN they were considered to MATCH; when they clustered into multiple BINs unique to that species they were SPLIT (e.g. revealing cryptic genetic diversity); a species placed in a single BIN together with individuals of another species found only in that BIN constituted a MERGE (e.g. revealing species indistinguishable through barcodes); and species with complex partitioning involving both a merge and a split fell into the MIXTURE category (e.g. revealing potential misidentification or hybridization issues). Identifications of non-native and invasive specimens were inferred on the basis of their BIN assignments. Identifications were considered successful when they clustered within a “MATCH” or “SPLIT” BIN that contained expert-identified reference specimens.

## Results

DNA was extracted from 140 channid specimens, 121 of which generated high quality barcode sequences, yielding coverage for a total of 25 of the 36 (Table 1, Table S1, Figure S1) described species of snakeheads. Another 129 GenBank sequences were also included, for a combined analysis of 250 specimens (DOI website. Available: dx.doi.org/10.5883/DS-DSCHA. Accessed 2014 May 21) (Table S1). Each species was represented by between 1 to 35 individuals with sequences ranging from 561 to 666 base pairs in length. No indels or stop codons were detected during sequence alignment, suggesting the absence of pseudogenes. Mean nucleotide frequencies across all sequences were T = 27.9%, C = 30.2%, G = 18.2%, A = 23.7%.

**Figure 1:**
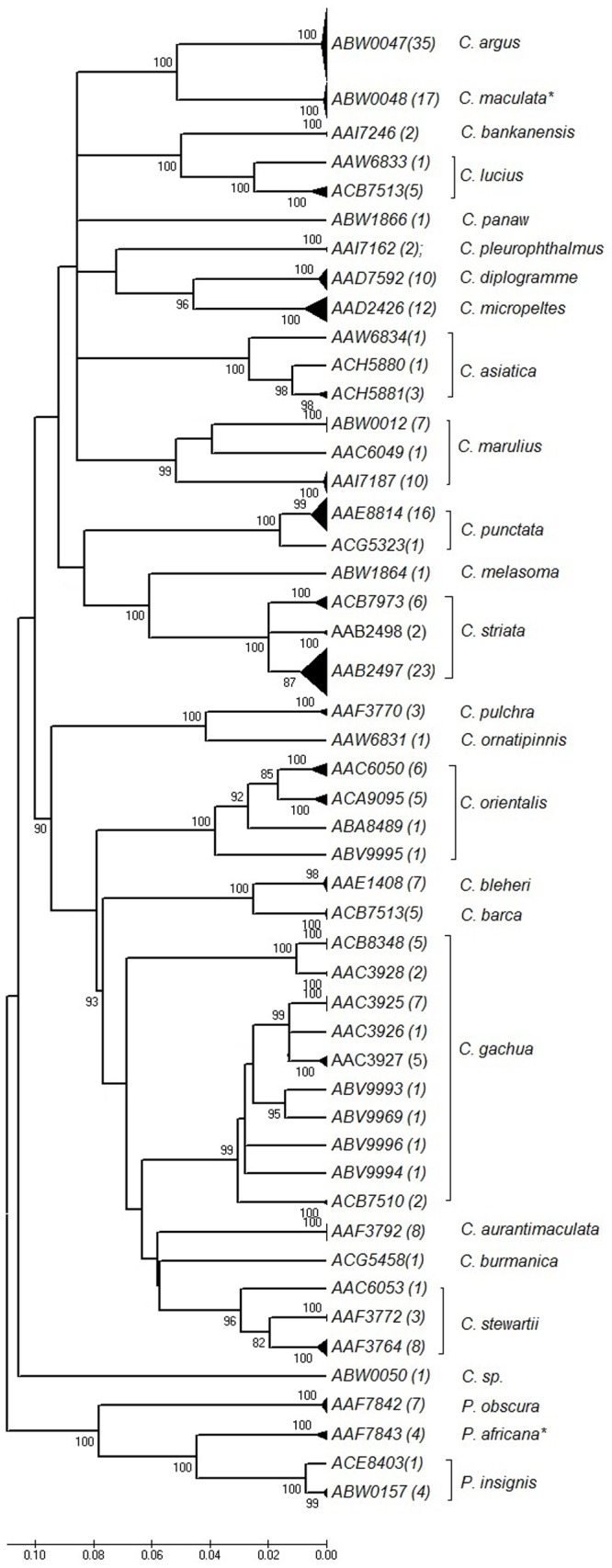
Collapsed Funnel Diagram of Neighbour Joining Tree displaying Intraspecific Variation within Channidae. Triangle bars represent genetic diversity with Channidae. Numbers contained in bracket of each species represent number of individuals that clustered in that particular haplogroup. The species that showed phylogeographical structuring had localities listed after the semi-colons within that particular haplogroup. Species C. maculata* is represented by nine C. sp., one *C. maculata*, six *C. argus*, one *C. argus (male)x C. maculata* (female). Species P. africana* is represented by three *P. africana* and one *P. obscura*.

Of the 25 species represented by the 250 individuals, 14 species represented a MATCH (one individual was only named to genus and not included in this number), 9 represented a SPLIT, and 2 species represented a MIXTURE (Table 2). These 25 species partitioned into 49 BINs, 19 of which were represented by singletons. The average intraspecific variation for the *Parachanna* genus was 0.43% and for the *Channa* genus was 1.58% (Figure 1, Table 2; using species as categories, not BINs). The species that represented MATCHes, for which more than one sequence per species was available, exhibited mean intraspecific divergences of 0.16% with a range from 0% to 0.37% (Table 2). Two species, *P. africana* and *C. maculata* represent a MIXTURE, in which *P. obscura* and *C. argus* individuals, respectively, group within them. Additionally, of the species that constituted MATCHes, four named species (individual named to genus not included since identity is unknown) were represented by singletons and could not be assessed for intraspecific diversity.

**Table 2:** List of BOLD species in DS-DSCHA, their corresponding BIN categories, BIN numbers and individuals per BIN, intraspecific variations within the BIN and species, and the countries that the reference specimen and introduced specimens were sourced from for that particular BIN.

Species	BIN Category	BIN Number(s) (# of specimens)	Intraspecific variation			Reference Country	Detected In
*C. argus*	MATCH	ABW0047(35)	0.31%			China	USA
*C. asiatica*	SPLIT	AAW6834(1)	N/A		2.96%	Unknown	N/A
		ACH5880 (1)	N/A			China	
		ACH5881(3)	0.5%			China	
*C. aurantimaculata*	MATCH	AAF3792 (8)	0.05%			India	USA
*C. bankanensis*	MATCH	AAI7246 (2)	0%			Unknown	N/A
*C. barca*	MATCH	ACB7513(5)	0.07%			India	N/A
*C. bleheri*	MATCH	AAE1408 (7)	0.09%			India	N/A
*C. burmanica*	MATCH	ACG5458(1)	N/A			Myanmar	N/A
*C. diplogramma*	MATCH	AAD7592 (10)	0.26%			India	N/A
*C. gachua*	SPLIT	ABV9993 (1)	N/A		7.43%	Thailand	N/A
		ABV9969 (1)	N/A			Thailand	N/A
		ABV9996 (1)	N/A			Thailand	N/A
		AAC3926 (1)	N/A			Myanmar	N/A
		ABV9994 (1)	N/A			Thailand	N/A
		AAC3925 (7)	0%			Myanmar & Thailand	N/A
		AAC3927 (5)	0.22%			Thailand	N/A
		AAC3928 (2)	0%			India	N/A
		ACB7510 (2)	0.17%			Indonesia	N/A
		ACB8348 (5)	0%			India	N/A
*C. species*	MATCH	ABW0050 (1)	N/A			Indonesia	N/A
*C. lucius*	SPLIT	AAW6833 (1)	N/A	2.06%		Malaysia	N/A
		ABW0051 (5)	0.63%			Malaysia & Thailand	N/A
*C. maculata*	MIXTURE	ABW0048 (17)	0.02%			Vietnam, China	Canada & USA
*C. marulius*	SPLIT	ABW0012 (7)	0%	5.80%		N/A	Canada & USA
		AAI7187 (10)	0.12%			India	N/A
*C. cf. marulius*		AAC6049 (1)	N/A			Unknown	N/A
*C. melasoma*	MATCH	ABW1864 (1)	N/A			Singapore	N/A
*C. micropeltes*	MATCH	AAD2426 (12)	0.28%			Thailand	Canada & USA
*C. orientalis*	SPLIT	ABV9995 (1)	N/A	3.35%		India	N/A
		AAC6050 (6)	0.65%			India & Myanmar	N/A
		ACA9095 (5)	0.46%			India	N/A
		ABA8489 (1)	N/A			India	N/A
*C. ornatipinnis*	MATCH	AAW6831 (1)	N/A			Unknown	N/A
*C. panaw*	MATCH	ABW1866 (1)	N/A			N/A	Denmark
*C. pleurophthalmus*	MATCH	AAI7162 (2)	0%			Unknown	N/A
*C. pulchra*	MATCH	AAF3770 (3)	0.37%			Unknown	N/A
*C. punctata*	SPLIT	AAE8814 (16)	0.65%	0.92%		India, Myanmar	N/A
		ACG5323(1)	N/A			Myanmar	N/A
*C. stewartii*	SPLIT	AAF3764 (8)	0.42%	2.53%		India	N/A
		AAF3772 (3)	0%			India & Thailand	N/A
*C. cf. stewartii*		AAC6053 (1)	N/A			Unknown	N/A
*C. striata*	SPLIT	AAB2497 (23)	0.62%		1.98%	Indonesia, Thailand	Philippines & USA
		ACB7973 (6)	0.29%			India (Assam)	N/A
		AAB2498 (2)	0.18%			India (Tamil Nadu)	N/A
*P. africana*	MIXTURE	AAF7843 (4)	0.48%			Democratic Republic of the Congo	N/A
*P. insignis*	SPLIT	ABW0157 (4)	0.09%		0.63%	Democratic Republic of the Congo, Republic of the Congo	N/A
		ACE8403(1)	N/A				
*P. obscura*	MATCH	AAF7842 (7)	0.18%			Nigeria, Senegal	N/A

Note: For species *P. africana*, intraspecific variation was calculated removing specimen identified as *P. insignis*, NRSC039-11.

**Figure 2:**
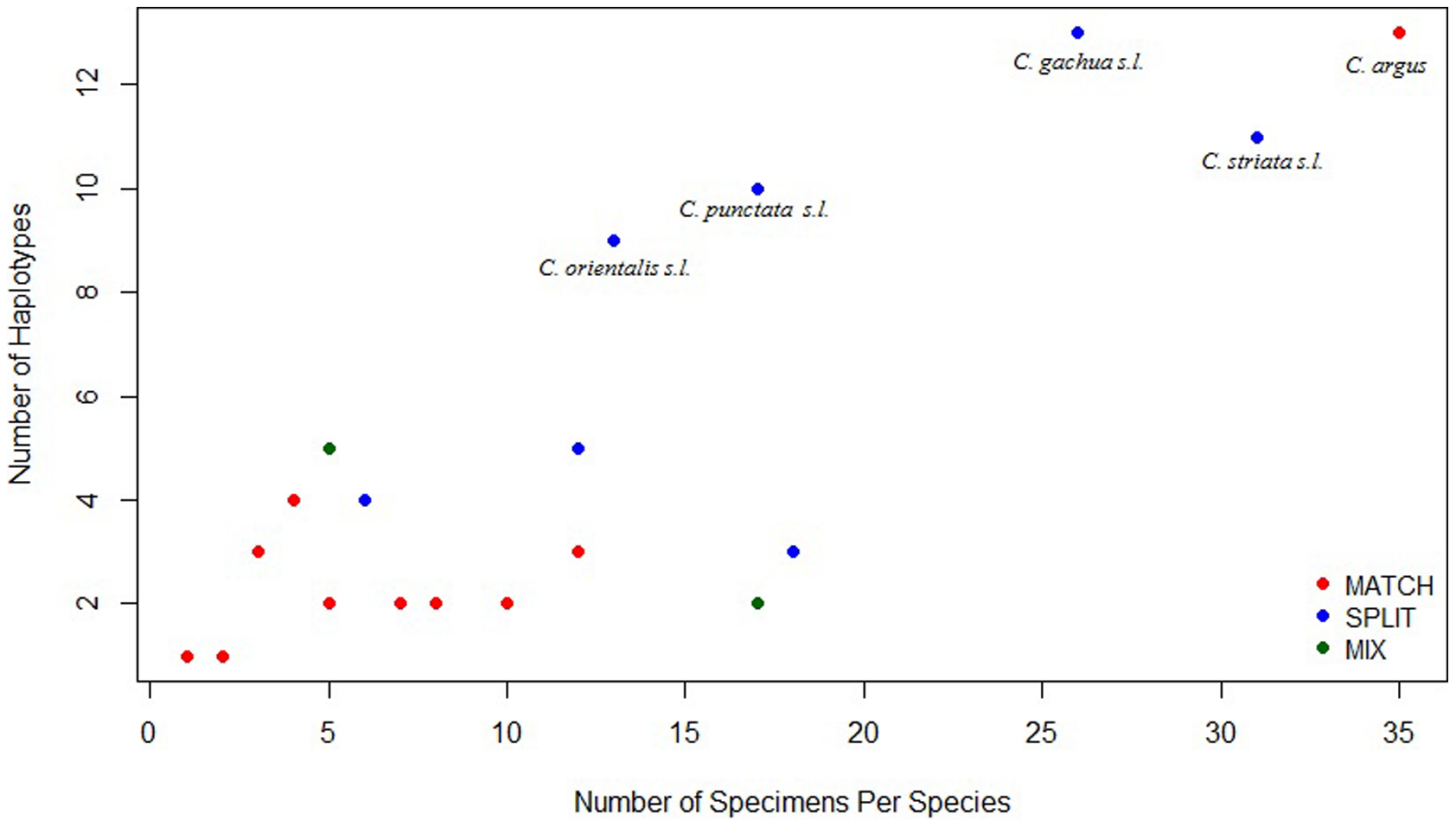
Scatterplot of Number Specimens in a Species against Number of Haplotypes. Red dots represent MATCH species, blue dots represent SPLIT species, Green dots represent MIX species. Species written on the Figure represent “outliers”, which constitute species with high number of specimens and high numbers of haplotypes.

To decipher if there was a relationship between BIN partitioning and sample size, a graph of number of specimens per species against number of unique haplotypes was generated (Figure 2). The species that were “outliers,” (i.e./high sampling effort and high unique haplotypes) consisted of *C. argus* (MATCH) and *C. orientalis, C. punctata, C. gachua and C. striata* (SPLIT). Three of the aforementioned SPLIT snakehead species are recognized as potentially harbouring cryptic diversity according to the literature. Five MATCH species had a high number of barcodes (≥5) and low number of unique haplotypes (≤3), suggesting there is no obvious relationship between BIN partitioning and sample size.

### SPLITS: cryptic diversity within phenotypic species

Substantial intraspecific divergences were observed among sequences from specimens morphologically identified as *C. striata, C. marulius*, and *C. gachua*, which exhibited intraspecific divergences of 1.98%, 5.8%, and 7.43% respectively (Figure 1; Table S1, Table 2). These species, as currently circumscribed, are suspected of harbouring cryptic diversity according to the literature, and our results indicate high levels of intraspecific variation [2]. *C. striata* was comprised of three distinct genetic clusters (BINs) sourced from the following regions: China, Indonesia and Thailand (and North American introductions) (23 individuals; BIN AAB2497), North East India (6 individuals; BIN ACB7973), and South India (2 individuals; BIN AAB2498). The average internal p-distance values of the three BINs were 0.36%, and these three BINs differed from each other by a mean p-distance of 4.2% (range of 3.9%-4.4%). Sequence data from *C. marulius* specimens yielded two distinct clusters: the reference individuals collected from India (10 individuals; BIN AAI7187) had a mean internal p-distance of 0.12%, and the non-native specimens obtained from the established population in Florida and from a pet store in Windsor (7 individuals; BIN ABW0012) had an internal p-distance of 0%. The two *C. marulius* BINs differed from each other by 10.4%. *C. gachua* showed the highest amount of intraspecific variation of all species in this study. It consists of ten divergent BINs sourced from a wide geographic distribution spanning India, Indonesia, Myanmar, and Thailand (Table 2), five of which occurred as singletons in the dataset. Each of these species exhibited phylogeographic structuring, as the divergent BINs within each putative taxon corresponded to a specific geographic region. Lastly, *C. punctata* harbours cryptic diversity with two BIN clusters (16 individuals; BIN AAE8814 and 1 individual; BIN ACG5323) with a mean internal distance of 3.2% between these two BINs. By contrast, although *C. micropeltes* is believed to potentially contain cryptic diversity [2], barcode sequences from Thailand formed a single distinct cluster, with mean intraspecific divergences of 0.28%. Species C. *asiatica* (BINs: AAW6834, ACH5880, ACH5881), *C. lucius* (BINs: AAW6833, ABW0051), *C. orientalis* (BINs: ABV9995, AAC6050, ACA9095, ABA8489), *C. stewartii* (BINs: AAF3764, AAF3772), *P. insignis* (BINs: ABW0157, ACE8403) each represented a SPLIT: although not considered as species complexes in the literature, the first four taxa exhibited moderate genetic diversity with mean internal divergence values of 2.96%, 2.01%, 3.60%, 1.80%, respectively, while that of *P. insignis* was more typical of intraspecific variation at 0.52%.

**Figure 3:**
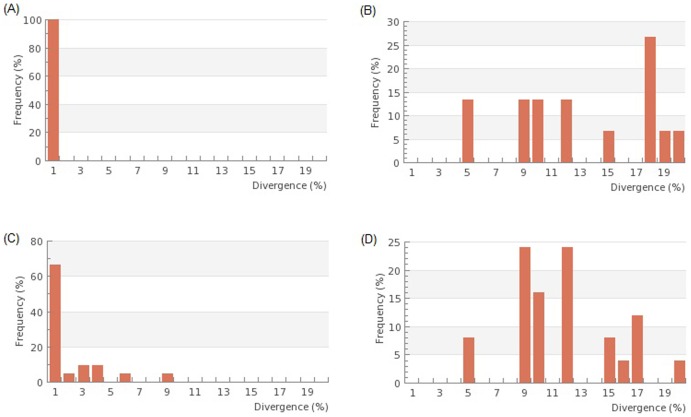
Bar graphs representing sequence diversity within and between species. (A) Mean intraspecific divergences within species that constitute a MATCH (B) Distance to nearest neighbour between species that constitute a MATCH (C) Mean intraspecific divergences within all species (minus potential misidentifications, hybrids, and individuals named only to genus) (D) Distance to nearest neighbour between all species (minus potential misidentifications, hybrids, and individuals named only to genus).

A graph comparing mean intraspecific divergences (Figure 3A) and distance to nearest neighbour (Figure 3B) of MATCH species reveals that the mean intraspecific variation is lower than the distance to the nearest neighbour. A graph comparing all snakehead species mean intraspecific divergence (Figure 3C) and distance to nearest neighbour (Figure 3D) does not reveal such clear separation. However, the figures make evident what may be species complexes as shown by the large amounts of intraspecific variation.

### MIXTURE: challenges for taxonomic resolution

Sequence data for specimens contained in BIN ABW0048 highlighted ambiguities in morphological identification and/or potential limitations of barcoding as a maternally inherited marker. For example, BIN ABW0048 consisted of 17 individuals; nine *C. sp*., one *C. maculata*, six *C. argus*, one *C. argus (male) x C. maculata (female)* (Figure 1), and therefore result in multiple species sharing the same BIN. The presumptive reference DNA barcodes for *C. argus* were obtained from sequences deposited on GenBank for individuals sourced from China (Zhou et al unpublished, [36], [37]), while the *C. maculata* reference specimens were sourced Vietnam (NRSC042-11; [38]) and China ([36], [37], [39]; Zhu et al. 2013, Wang et al. 2013) (see *C. maculata*; Table 3). The *C. argus* specimen whole mitochondrial genome sequence [40] clustered in the same BIN (ABW0048) as the *C. maculata* and *C. maculata* (F) x *C. argus* (M) hybrid mitochondrial genome sequences of specimens [36], [39], [40], and therefore highlights the difficulties with identification of this species.

**Table 3:** Introduced snakehead specimens with corresponding matches on BOLD to reference specimens.

Species Name	BOLD Sample ID	BOLD Process ID	BIN	Locality	Barcode Library Reference Match
*C. argus*	NRS025	NRSC025-11	ABW0047	New York (Market)	Genbank: *C. argus* JQ358715-19, JX978724, GBGCA4826-13- GBGCA4836-13
	MCsn4	DSCHA069-12	ABW0047	New York (Water)	
	MCsn3	DSCHA068-12	ABW0047	New York (Water)	
	NRS050	NRSC050-12	ABW0047	Pennsylvania (Water)	
	NRS065	DSCHA043-12	ABW0047	New York (Water)	
	NRS006	NRSC006-11	ABW0047	Virginia (Water)	
	NRS005	NRSC005-11	ABW0047	Virginia (Water)	
	NRS063	DSCHA041-12	ABW0047	New York (Water)	
	MC2sn1	DSCHA066-12	ABW0047	New York (Water)	
	MCsn6	DSCHA071-12	ABW0047	New York (Water)	
	MCsn5	DSCHA070-12	ABW0047	New York (Water)	
	NRS026	NRSC026-11	ABW0047	New York (Market)	
	NRS057	NRSC057-12	ABW0047	Pennsylvania (Water)	
	NRS024	NRSC024-11	ABW0047	New York (Market)	
	MCsn7	DSCHA072-12	ABW0047	New York (Water)	
	NRS004	NRSC004-11	ABW0047	Virginia (Water)	
	MCsn2	DSCHA067-12	ABW0047	New York (Water)	
	NRS062	DSCHA040-12	ABW0047	New York (Water)	
*C. aurantimaculata*	NRS028	NRSC028-11	AAF3792	North Carolina (Aquarium)	Genbank: *C. aurantimaculata* HM117172-76, EU342193, EU342194
*C. marulius*	NRS001	NRSC001-11	AAI7187	Ontario (Aquarium)	No matches
	NRS051	NRSC051-12	AAI7187	Florida (water)	
	NRS055	NRSC055-12	AAI7187	Florida (water)	
	NRS056	NRSC056-12	AAI7187	Florida (water)	
	NRS054	NRSC054-12	AAI7187	Florida (water)	
	NRS052	NRSC052-12	AAI7187	Florida (water)	
	NRS053	NRSC053-12	AAI7187	Florida (water)	
*C. micropeltes*	NRS002	NRSC002-11	AAD2426	Ontario (Aquarium)	BOLD project sample ID: CHAN031-32, CHAN020, 021, 023, 024 Other BOLD project:CHMI-Petshop-1, CHMI-Petshop-2
	NRS029	NRSC029-11	AAD2426	Virginia (Aquarium)	
	NRS030	NRSC030-11	AAD2426	Virginia (Aquarium)	
	CM_Quebec	DSCHA074-13	AAD2426	Toronto (Aquarium)	
*C. panaw*	NRS041	NRSC041-11	ABW1866	Introduced in Denmark	No match
*C. sp.*	BCsnakehead8	DSCHA064-12	ABW0048	British Columbia (market)	Genbank: *C. argus* whole mitogenome NC-015191, KC310861, JX978724, *C argus x C. maculata* hybrid JX978725, GBGCA4825-13. BOLD project sampleID:*C. maculata* NRS042,
	BCsnakehead5	DSCHA061-12	ABW0048	imported SFU from China	
	BCsnakehead1	DSCHA057-12	ABW0048	imported SFU from China	
	BCsnakehead4	DSCHA060-12	ABW0048	imported SFU from China	
	BCsnakehead2	DSCHA058-12	ABW0048	imported SFU from China	
	BCsnakehead3	DSCHA059-12	ABW0048	imported SFU from China	
	BCsnakehead7	DSCHA063-12	ABW0048	British Columbia (Market)	
	BC-lagoon	DSCHA065-12	ABW0048	British Columbia (Wild)	
	BCsnakehead6	DSCHA062-12	ABW0048	British Columbia (Market)	
*C. maculata*	NRS027	NRSC027-11	ABW0048	North Carolina (Water)	
	NRS007	NRSC007-11	ABW0048	British Columbia (Market)	
*C. striata*	NRS031	NRSC031-11	AAB2497	United States (Market)	Genbank: JQ661365-68,Other BOLD project sampleID: Cstr1-5, Cstri1-LdB-Cstri5-LdB, JQ661364, GBGCA4818-13- GB, GCA4820-13 BOLD project sampleID: CHAN003, NRS011, NRS018,
	NRS032	NRSC032-11	AAB2497	United States (Market)	

### Assignment of non-native specimens to BINs

The barcode reference library was used to assess the identity of the unknown snakehead species based on shared BIN membership with an expert-identified reference specimen (Table 3). The non-native species *C. panaw* (NRSC041-11) and *C. marulius* (NRSC001-11, NRSC051-11-NRSC056-11) did not match any of the BINs populated by reference specimens, representing new BINs to BOLD. Hence barcoding cannot currently be used to identify them, but their barcodes can serve as being representative of these morphospecies until more definitive sequences can be derived from vouchered specimens of known provenance. Barcode sequences from the other morphologically identified invasive specimens matched their corresponding BIN reference DNA barcodes; C. *argus* from New York (NRSC024-11, NRSC025-11, DSCHA066-12-072-12, 040-12, 041-12, 043-12), Pennsylvania (NRSC050-12, NRSC057-12), and Virginia (NRSC004-11-0006-11) had a 0.3%, 0.4% and 0.4% genetic distance to corresponding reference sequences (BIN: ABW0047). *C. aurantimaculata* confiscated from an aquarium (NRSC028-11) had a 100% sequence identity to corresponding reference sequences (BIN: AAF3792), while *C. micropeltes* from petshops (NRSC002-11, NRSC029-11-30-11, DSCHA074-13) exhibited 0.4% genetic distance to corresponding references (BIN: AAD2426). Lastly, *C. striata* (NRSC031-11-32-11) exhibited a 1.6% genetic distance to all corresponding *C. striata* sequences (BIN AAB2497, ACB7973, AAB2498; Table 3), but grouped into BIN AAB2497. The British Columbia (BC) wild-caught specimen (DSCHA065-12), BC market specimens (NRSC007-11, DSCHA062-12-064-12), and Chinese imported specimens from BC (DSCHA057-12-DSCHA061-12) were originally identified only to genus (*Channa*; D. Scott, pers. comm.) based on morphology. When tested against the DNA barcode reference library, they were identified as *C. maculata* (or *C. argus x maculata* hybrid with *C. maculata* maternal parent) (Table 3), and with the exception of specimen NRSC042-11 (BIN: ABW0048) differing by one base pair in the barcode region, all other reference specimens contained the same barcode. The Lake Wylie, North Carolina specimen (NRSC027-11) was originally identified as *C. argus* but had a 100% sequence identity to reference sequences for *C. maculata* (with the exception of one base pair difference for specimen NRSC042-11), and was subsequently re-identified morphologically as *C. maculata* (BIN: ABW0048) (W. Starnes, North Carolina Museum of Natural Sciences, pers. comm.). This case highlights the value of barcoding in flagging potential cases of misidentification.

## Discussion

This study represents the largest and most comprehensive global synthesis of sequence diversity within the family Channidae yet undertaken. In lieu of limited snakehead taxonomic expertise and inadequate morphological keys, molecular techniques provide a rapid method of identification. The substantial sequence diversity identified in this study within broadly defined taxa in both channid genera highlights the need for comprehensive examination of the molecular and morphological systematics within the Channidae. It also highlights the need for integrative taxonomic resolution and delineation of species boundaries. This study is constrained by limited representation from source populations and type localities for described species e.g. [41], as well as incomplete representation of species from the genus *Channa*. At least nine described species have yet to be added to the BOLD database, and nine of the *sensu lato* species included in this study may account for undescribed cryptic taxa as revealed by the barcode data and related BIN assignments. Based on the biogeographic range and distribution of snakehead species and genera, it seems apparent that substantial genetic and evolutionary diversity within the Channidae remains to be described.

The results substantiate a growing body of work indicating that DNA barcodes can be used to discriminate between various channid species, shown to be effective in other fish studies [18], [26], [42]. Past studies focused on a relatively small number of snakehead species occurring within a particular region [12], [14], [25]. Efforts to characterize the genetic diversity of snakeheads have been restricted [16], [27], [38]. The present study has attempted to overcome this limitation by adding to the barcode library for snakeheads and providing novel coverage for 25 species. Importantly, the sequences compiled from this study also include additional metadata concerning collection locality, digital images of the vouchers and metadata pertaining to them, electropherogram “trace” files and PCR primer sequences where possible, which enhances their fitness-for-use in molecular diagnostic applications (as discussed in [18]). With this contribution, we also make a plea for other researchers to not only include the COI barcode as a common marker in their studies, but to also adhere to barcode data standards for reporting provenance data.

### Delineation and species identification using Barcodes and BINS

DNA barcoding proved to be an effective tool for species-level identification of snakeheads. Of the 25 species that were presented in this study, 14 were a MATCH, reinforcing the finding that individuals of the same species tend to possess diagnostic barcode arrays [43]. Nine species were SPLIT across multiple BINs, highlighting the existence of discrete phylogeographically structured barcode clusters separated by genetic distances typically associated with different species and suggesting the presence of possible cryptic species. Two species were a MIXTURE, indicating that there are ambiguities in identification and cases of hybridization. Our efforts to expand barcode coverage for the channid contribute significantly to a synthesis that includes 70% of their described species diversity (25 of 36 spp.). The barcodes segregate into 49 BINs, suggesting that our current taxonomic framework under-represents the genetic diversity of the group, a finding consistent with other large-scale barcode surveys of freshwater fishes [21], [44].

The *Parachanna* species have overlapping ranges and are morphologically very similar. This renders identification very difficult, as indicated by the assignment of *P. africana* and *P. obscura* under BIN AAF7843, which constitutes a MIXTURE. Future efforts should be aimed at sourcing more specimens and carefully examining the taxonomy of this group.

In addition to this study, the BIN assignment and classification system has been successfully implemented in other model organisms such as spiders [45] and moths [46]. In well-studied species and taxonomic groups, the BIN system is ideal because it allows for unambiguous classification by using MATCH categories. In taxonomic groups that have been incompletely resolved, classification can be more difficult as a barcoding gap may not be present between recently diverged species, while older species could be represented by artificial splits [47]. While the BIN algorithm shows considerable promise, it should also be implemented with caution and in concert with existing taxonomic and phyletic information wherever possible.

### Identification of cryptic species and hybridization

The literature suggests that several snakehead species as currently circumscribed could each constitute species complexes in their own right [2], [27], [38]. If true, the species in question could represent a SPLIT, and would therefore partition into multiple BINs as a result of high intraspecific variation. This is seen for *C. gachua*, *C. marulius, C. punctata* and *C. striata*, which are characterized by SPLITs, and partitioned into ten, two, two and three BINs respectively, suggesting that they harbour cryptic diversity (Table 2). Of particular importance to note is that BINs show phylogeographic structuring making them particularly interesting for inferring introduction pathways involving broadly distributed morphospecies (e.g. like many of the taxon concepts currently used for snakeheads).

Hybrids cannot be detected with DNA barcoding directly because mitochondrial DNA is typically only inherited from the maternal parent. Adamson et al. [38] sequenced nuclear gene RP1 and suggested that *C. striata* is a product of genetic introgression rather than speciation. If *C. striata* specimens could be extensively sampled throughout the species range, this could be very helpful to aid in identifying invasion sources and expansion pathways, as this species is phylogeographically structured [48], [49]. For example, *C. striata* (NRSC031-11, 032-11; Table S1) were sourced from a market in New York but based on the observed sequence similarity, we can predict that these specimens most likely originated from Southeast Asia as opposed to India, although geographic representation (sampling intensity) for this region was limited.

Genetic diversity in *C. gachua*, *C. marulius*, and *C. punctata* has been largely unexplored. Low amounts of intraspecific diversity were observed in prior studies [14], [27], [38], but this could reflect a limited scope of geographical sampling. In contrast, our study partitions *C. gachua* into ten BINs suggesting cryptic diversity with the further acknowledgement that half of these clusters are represented by a singleton specimen. Adamson et al. [38] suggested *C. marulius* might harbour cryptic diversity and observed that the *C. marulius* sourced from India was genetically divergent from the *Channa* that was only identified to genus sourced from Cambodia, but consistently grouped together in a phylogenetic analysis. The results from our study indicated that the *C. marulius* Indian haplogroup partitions into a separate BIN from the North American *C. marulius* population, ruling out the Indian population as a source of invasion. In the case of *C. punctata*, previous studies using RAPD and allozymes suggested that geographic distance is positively correlated with genetic diversity within this species [50], [51]. Our study revealed two BIN clusters (AAE8814, ACG5323), yet all but one sequence segregated into one haplogroup. Specimen availability was limited for this species, and a broader geographic coverage would likely reveal additional haplotypes. This particular species has achieved a low-risk “near-threatened” status [52]. While snakeheads are viewed as “invasive”, this particular species is declining as a result of over-exploitation, disease and habitat loss [8], [48], suggesting that conservation efforts need to be focused on their preservation.

Believed to possess cryptic diversity, *C. micropeltes* showed very low intraspecific variation and formed only one BIN in our study. These results are unsurprising; as we only had access to samples from Thailand, despite the fact that *C*. *micropeltes* as currently circumscribed is thought to occupy a much larger geographic range. Additional sampling for *C. micropeltes* is likely to reveal higher intraspecific mtDNA variation similar to that observed in other channid species [50], [51].

### Applications for species introductions and invasions

A key component of conservation management is the identification of non-native and potentially invasive species. Consequently one objective of this study was to resolve the taxonomic identity of introduced snakeheads. This was accomplished by examining the non-native snakehead species BINs assignments to see if they cluster with expert-identified reference specimens used to construct the barcode library and perhaps shed light on potential expansion or invasion pathways. While this was successfully assessed for most introduced snakeheads (Table 3), there remains ambiguity with respect to the identity of the United States species (*C. argus, C. maculata, C. marulius*). While *C. marulius* shows a broad distribution in southern and south-eastern Asia (Table 1), individuals sourced from their native ranges were only available from India for this study [52] and these specimens were genetically divergent from individuals found in North America. The barcode reference library remains incomplete for this species, and specimens need to be sampled throughout its native range before the *C. marulius* source of origin can be established.

Difficulties also lie in accurate identification between *C. argus* and *C. maculata*, as these two species names have often been interchanged [2], [37]. Most recently, Wang and Yang [40] sequenced the whole mitogenome of *C. argus*, but typical of most mitogenome sequences deposited in GenBank, they made no reference to a voucher specimen making it difficult to corroborate their identification. A subsequent study conducted by Zhu et al. [36] in which whole mitogenomes were sequenced for *C. argus, C. maculata*, and a hybrid *C argus* (male) x *C. maculata* (female), showed contradictory results. Zhu et al. [36] observed that the *C. argus* specimen sequenced by Wang and Yang [39] shared the same DNA barcode sequence as their *C. maculata* and hybrid haplogroup, suggesting the putative *C. argus* material sequenced by Wang and Yang [40] could have been derived from a misidentified specimen of *C. maculata* or an unrecognized hybrid. The *C. maculata* reference specimen sourced for our study was obtained from Vietnam [38] and shares the same BIN with *C. maculata* and a hybrid [36], [38] as well as a likely misidentified *C. argus* [40].

A second case of mistaken identity was observed with the Lake Wylie, North Carolina specimen (NRSC027-11) that was originally identified as *C. argus*. The morphological voucher specimen was retained and later re-identified as *C. maculata* as comparisons with barcode reference sequences alerted to that possibility (per comm. W.C. Starnes, NCSMNS.). The remaining northern snakehead specimens sourced from Virginia, Pennsylvania, and New York matched *C. argus*. Recent literature [36], [37], however, suggests that *C. argus* (female) and *C. maculata* (male) have been crossed for aquaculture purposes. If such hybrids are fertile, genetic introgression could limit barcode identification to the level of species-pair for this group.

As management decisions are influenced by the perceived biological attributes of the species that are thought to be present, accurate species identification is critical. For example, *C. argus* is a temperate, cold-tolerant species that has a much broader environmental suitability throughout North America than the tropical *C. maculata* [5], [53] and could therefore call for differing levels of intervention. Specimens that were purchased from a fish market in BC, Canada (NRSC007-11, DSCHA062-11-064-11) grouped with *C. maculata* [54], which was surprising as it was thought that *C. argus* represents the only species being imported into BC [52], [53]. The BC wild-caught specimen (DSCHA065-12) also grouped with the *C. maculata* haplogroup and could potentially represent either *C. maculata* or a hybrid with *C. maculata* as maternal parent species [50]. The hybrid species could pose its own threats because it is able to grow very fast; it has higher survival rates and is better able to endure stress than its parent species [55]. Regardless, this example illustrates how a DNA barcode reference library can provide insight into past invasions and shed new light on emerging invasion threats, calling for continued efforts to populate it and perhaps extend coverage by including a nuclear marker to aid the identification of hybrids.

Although *C. maculata* has been established in Hawaii for over a century [2], this population is a prime example of taxonomic misidentification. Originally believed to represent *C. striata*, further examination [7] revealed it to be *C. maculata*. Courtenay et al. [7] argue there is a need for accurate snakehead identification in order to make practical predictions about their effects in non-indigenous environments. They also state that there is a need to acquire voucher specimens so that re-examination can be possible.

An emerging potent molecular tool that is being applied to aquatic invasive species monitoring is environmental DNA (eDNA) detection. This approach employs the presence of species-specific DNA sequence motifs using genomic DNA extracts from water samples [56]. Because aquatic organisms shed DNA into their environment, it can be applied to infer species presence and has been used successfully to detect invasive species, even at low abundances [57], [58]. The sequence data from this work and similar studies could inform the development of species-specific PCR primer and probe sets for the detection of eDNA for species such as *C. argus, C. marulius*, and *C. maculata*. As a caveat, the large number of BINs documented in this study, despite the relatively low numbers of available samples, indicates that there is substantial genetic diversity within and among snakehead species that has yet to be documented. Accordingly, eDNA surveillance would only be effective for detecting haplotypes that fit within the BINs/species that are present in the BOLD reference library. Hence, while it may be possible to build primers and probes for known haplotypes, the risk of generating false negative results from targeted marker development remains substantial for at least some members of this group.

## Conclusions

This study represents the most comprehensive account of mtDNA diversity within Channidae, and has contributed to the species diversity within this family. In order to better understand and delineate this fascinating group of species, efforts should be focused on more intensive sampling at hierarchical spatial scales, coupled with both morphological and genetic analysis. Similarly, this study also highlights the importance of documenting the barcode profiles of unknowns and non-native specimens. When placed in the BIN framework, these sequences provide additional evidence that the barcode reference sequence library remains incomplete as not all specimens' barcodes cluster with those of reference specimens. Moreover, their retention in the BOLD BIN schema suggests that as the reference sequence library grows, it may be possible to retrospectively identify their putative source population. Hence, we argue for continuing to not only query non-natives against the reference library but also deposit their sequences in it because they are important for documenting the extent of haplotypic diversity in nature and can help contribute to the creation of robust tools for detecting the eDNA of invasive species.

## Supporting Information

Figure S1 Neighbour Joining Tree of collapsed snakehead sequences with species name, process ID, sample ID and BIN number.
(PDF)

Table S1 Table of species name, process ID, sample ID, country specimen was sourced from, museum ID if applicable, if the specimen is from a native or non-native range and BIN number.
(DOC)
