# Supplementary material for: Calibrating Snakehead Diversity with DNA Barcodes: Expanding Taxonomic Coverage to Enable Identification of Potential and Established Invasive Species
Source: PLoS One. 2014 Jun 10;9(6):e99546. doi: 10.1371/journal.pone.0099546 (PMC4051700; doi:10.1371/journal.pone.0099546)
Supplement: Figure S1 — Neighbour Joining Tree of collapsed snakehead sequences with species name, process ID, sample ID and BIN number. (PDF) [file pone.0099546.s001.pdf]

BOLD TaxonID Tree

Title	:	Snakeheads [DS-DSCHA]
Date	:	7-December-2013
Data Type	:	Nucleotide
Distance Model	:	Pairwise Distance
Marker	:	COI-5P
Codon Positions	:	
Labels	:	SampleID, ProcessID, BIN uri
Filters	:	Length > 200
Colorization	:	[blue]=Stop Codons [red]=Contamination or misidentification

Sequence Count	:	250
Species count	:	29
Genus count	:	2
Family count	:	1
Unidentified	:	1

BIN Count	:	49

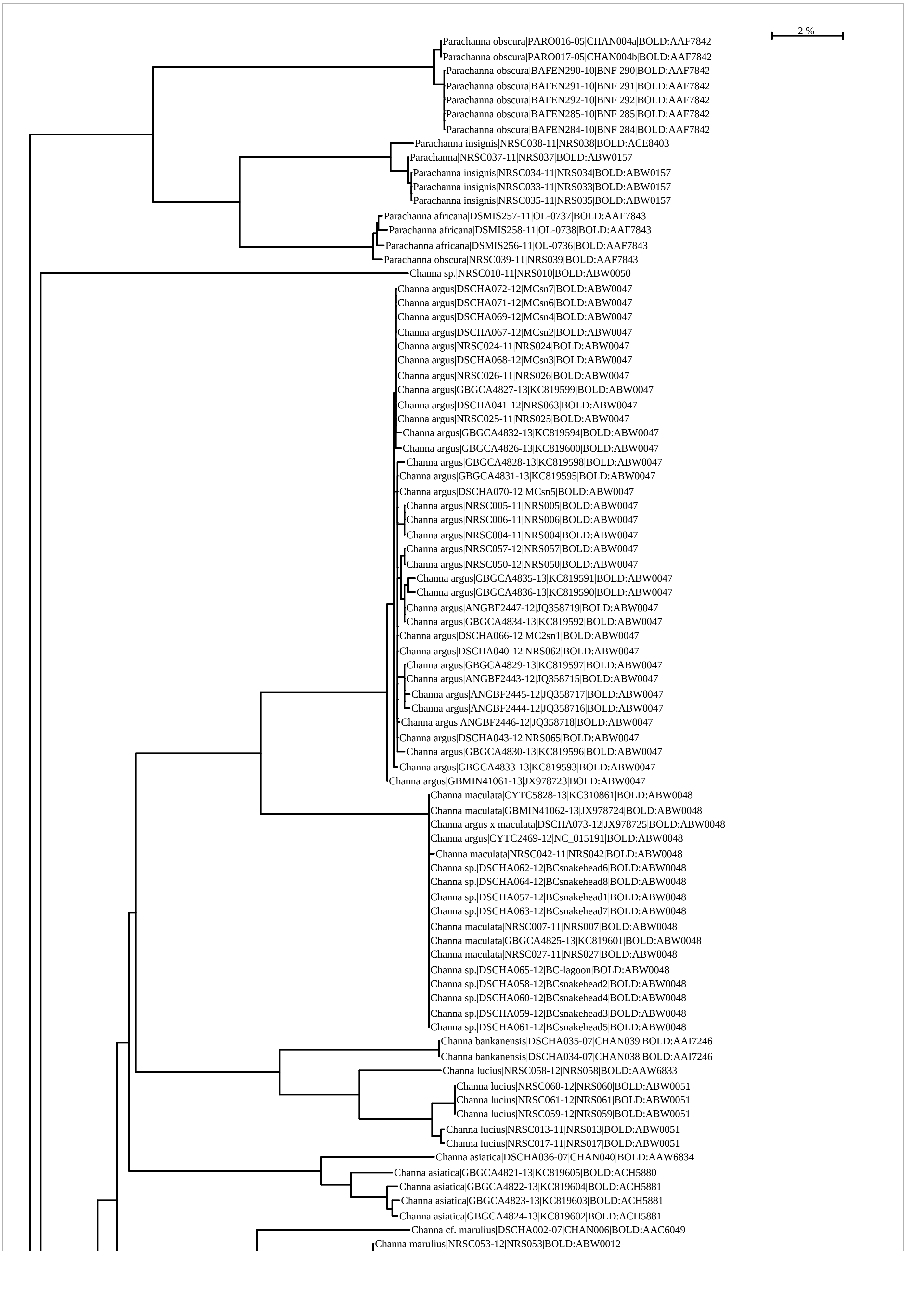
2 %
Parachanna obscura|PARO016-05|CHAN004a|BOLD:AAF7842
Parachanna obscura|PARO017-05|CHAN004b|BOLD:AAF7842
Parachanna obscura|BAFEN290-10|BNF 290|BOLD:AAF7842
Parachanna obscura|BAFEN291-10|BNF 291|BOLD:AAF7842
Parachanna obscura|BAFEN292-10|BNF 292|BOLD:AAF7842
Parachanna obscura|BAFEN285-10|BNF 285|BOLD:AAF7842
Parachanna obscura|BAFEN284-10|BNF 284|BOLD:AAF7842
Parachanna insignis|NRSC038-11|NRS038|BOLD:ACE8403
Parachanna|NRSC037-11|NRS037|BOLD:ABW0157
Parachanna insignis|NRSC034-11|NRS034|BOLD:ABW0157
Parachanna insignis|NRSC033-11|NRS033|BOLD:ABW0157
Parachanna insignis|NRSC035-11|NRS035|BOLD:ABW0157
Parachanna africana|DSMIS257-11|OL-0737|BOLD:AAF7843
Parachanna africana|DSMIS258-11|OL-0738|BOLD:AAF7843
Parachanna africana|DSMIS256-11|OL-0736|BOLD:AAF7843
Parachanna obscura|NRSC039-11|NRS039|BOLD:AAF7843
Channa sp.|NRSC010-11|NRS010|BOLD:ABW0050
Channa argus|DSCHA072-12|MCsn7|BOLD:ABW0047
Channa argus|DSCHA071-12|MCsn6|BOLD:ABW0047
Channa argus|DSCHA069-12|MCsn4|BOLD:ABW0047
Channa argus|DSCHA067-12|MCsn2|BOLD:ABW0047
Channa argus|NRSC024-11|NRS024|BOLD:ABW0047
Channa argus|DSCHA068-12|MCsn3|BOLD:ABW0047
Channa argus|NRSC026-11|NRS026|BOLD:ABW0047
Channa argus|GBGCA4827-13|KC819599|BOLD:ABW0047
Channa argus|DSCHA041-12|NRS063|BOLD:ABW0047
Channa argus|NRSC025-11|NRS025|BOLD:ABW0047
Channa argus|GBGCA4832-13|KC819594|BOLD:ABW0047
Channa argus|GBGCA4826-13|KC819600|BOLD:ABW0047
Channa argus|GBGCA4828-13|KC819598|BOLD:ABW0047
Channa argus|GBGCA4831-13|KC819595|BOLD:ABW0047
Channa argus|DSCHA070-12|MCsn5|BOLD:ABW0047
Channa argus|NRSC005-11|NRS005|BOLD:ABW0047
Channa argus|NRSC006-11|NRS006|BOLD:ABW0047
Channa argus|NRSC004-11|NRS004|BOLD:ABW0047
Channa argus|NRSC057-12|NRS057|BOLD:ABW0047
Channa argus|NRSC050-12|NRS050|BOLD:ABW0047
Channa argus|GBGCA4835-13|KC819591|BOLD:ABW0047
Channa argus|GBGCA4836-13|KC819590|BOLD:ABW0047
Channa argus|ANGBF2447-12|JQ358719|BOLD:ABW0047
Channa argus|GBGCA4834-13|KC819592|BOLD:ABW0047
Channa argus|DSCHA066-12|MC2sn1|BOLD:ABW0047
Channa argus|DSCHA040-12|NRS062|BOLD:ABW0047
Channa argus|GBGCA4829-13|KC819597|BOLD:ABW0047
Channa argus|ANGBF2443-12|JQ358715|BOLD:ABW0047
Channa argus|ANGBF2445-12|JQ358717|BOLD:ABW0047
Channa argus|ANGBF2444-12|JQ358716|BOLD:ABW0047
Channa argus|ANGBF2446-12|JQ358718|BOLD:ABW0047
Channa argus|DSCHA043-12|NRS065|BOLD:ABW0047
Channa argus|GBGCA4830-13|KC819596|BOLD:ABW0047
Channa argus|GBGCA4833-13|KC819593|BOLD:ABW0047
Channa argus|GBMIN41061-13|JX978723|BOLD:ABW0047
Channa maculata|CYTC5828-13|KC310861|BOLD:ABW0048
Channa maculata|GBMIN41062-13|JX978724|BOLD:ABW0048
Channa argus x maculata|DSCHA073-12|JX978725|BOLD:ABW0048
Channa argus|CYTC2469-12|NC_015191|BOLD:ABW0048
Channa maculata|NRSC042-11|NRS042|BOLD:ABW0048
Channa sp.|DSCHA062-12|BCsnakehead6|BOLD:ABW0048
Channa sp.|DSCHA064-12|BCsnakehead8|BOLD:ABW0048
Channa sp.|DSCHA057-12|BCsnakehead1|BOLD:ABW0048
Channa sp.|DSCHA063-12|BCsnakehead7|BOLD:ABW0048
Channa maculata|NRSC007-11|NRS007|BOLD:ABW0048
Channa maculata|GBGCA4825-13|KC819601|BOLD:ABW0048
Channa maculata|NRSC027-11|NRS027|BOLD:ABW0048
Channa sp.|DSCHA065-12|BC-lagoon|BOLD:ABW0048
Channa sp.|DSCHA058-12|BCsnakehead2|BOLD:ABW0048
Channa sp.|DSCHA060-12|BCsnakehead4|BOLD:ABW0048
Channa sp.|DSCHA059-12|BCsnakehead3|BOLD:ABW0048
Channa sp.|DSCHA061-12|BCsnakehead5|BOLD:ABW0048
Channa bankanensis|DSCHA035-07|CHAN039|BOLD:AAI7246
Channa bankanensis|DSCHA034-07|CHAN038|BOLD:AAI7246
Channa lucius|NRSC058-12|NRS058|BOLD:AAW6833
Channa lucius|NRSC060-12|NRS060|BOLD:ABW0051
Channa lucius|NRSC061-12|NRS061|BOLD:ABW0051
Channa lucius|NRSC059-12|NRS059|BOLD:ABW0051
Channa lucius|NRSC013-11|NRS013|BOLD:ABW0051
Channa lucius|NRSC017-11|NRS017|BOLD:ABW0051
Channa asiatica|DSCHA036-07|CHAN040|BOLD:AAW6834
Channa asiatica|GBGCA4821-13|KC819605|BOLD:ACH5880
Channa asiatica|GBGCA4822-13|KC819604|BOLD:ACH5881
Channa asiatica|GBGCA4823-13|KC819603|BOLD:ACH5881
Channa asiatica|GBGCA4824-13|KC819602|BOLD:ACH5881
Channa cf. marulius|DSCHA002-07|CHAN006|BOLD:AAC6049
Channa marulius|NRSC053-12|NRS053|BOLD:ABW0012

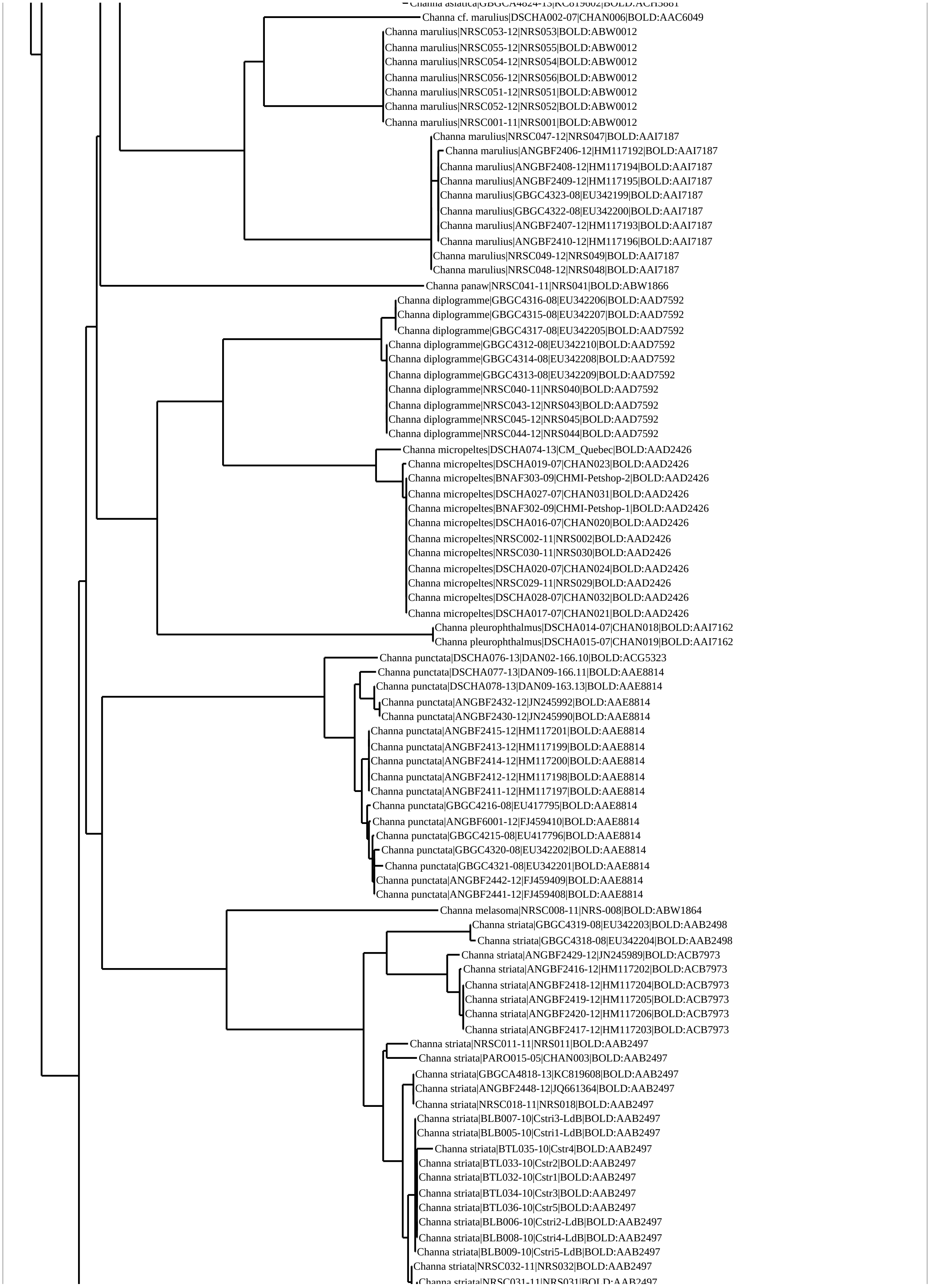

Channa cf. marulius|DSCHA002-07|CHAN006|BOLD:AAC6049
Channa marulius|NRSC053-12|NRS053|BOLD:ABW0012
Channa marulius|NRSC055-12|NRS055|BOLD:ABW0012
Channa marulius|NRSC054-12|NRS054|BOLD:ABW0012
Channa marulius|NRSC056-12|NRS056|BOLD:ABW0012
Channa marulius|NRSC051-12|NRS051|BOLD:ABW0012
Channa marulius|NRSC052-12|NRS052|BOLD:ABW0012
Channa marulius|NRSC001-11|NRS001|BOLD:ABW0012
Channa marulius|NRSC047-12|NRS047|BOLD:AAI7187
Channa marulius|ANGBF2406-12|HM117192|BOLD:AAI7187
Channa marulius|ANGBF2408-12|HM117194|BOLD:AAI7187
Channa marulius|ANGBF2409-12|HM117195|BOLD:AAI7187
Channa marulius|GBGC4323-08|EU342199|BOLD:AAI7187
Channa marulius|GBGC4322-08|EU342200|BOLD:AAI7187
Channa marulius|ANGBF2407-12|HM117193|BOLD:AAI7187
Channa marulius|ANGBF2410-12|HM117196|BOLD:AAI7187
Channa marulius|NRSC049-12|NRS049|BOLD:AAI7187
Channa marulius|NRSC048-12|NRS048|BOLD:AAI7187
Channa panaw|NRSC041-11|NRS041|BOLD:ABW1866
Channa diplogramme|GBGC4316-08|EU342206|BOLD:AAD7592
Channa diplogramme|GBGC4315-08|EU342207|BOLD:AAD7592
Channa diplogramme|GBGC4317-08|EU342205|BOLD:AAD7592
Channa diplogramme|GBGC4312-08|EU342210|BOLD:AAD7592
Channa diplogramme|GBGC4314-08|EU342208|BOLD:AAD7592
Channa diplogramme|GBGC4313-08|EU342209|BOLD:AAD7592
Channa diplogramme|NRSC040-11|NRS040|BOLD:AAD7592
Channa diplogramme|NRSC043-12|NRS043|BOLD:AAD7592
Channa diplogramme|NRSC045-12|NRS045|BOLD:AAD7592
Channa diplogramme|NRSC044-12|NRS044|BOLD:AAD7592
Channa micropeltes|DSCHA074-13|CM_Quebec|BOLD:AAD2426
Channa micropeltes|DSCHA019-07|CHAN023|BOLD:AAD2426
Channa micropeltes|BNAF303-09|CHMI-Petshop-2|BOLD:AAD2426
Channa micropeltes|DSCHA027-07|CHAN031|BOLD:AAD2426
Channa micropeltes|BNAF302-09|CHMI-Petshop-1|BOLD:AAD2426
Channa micropeltes|DSCHA016-07|CHAN020|BOLD:AAD2426
Channa micropeltes|NRSC002-11|NRS002|BOLD:AAD2426
Channa micropeltes|NRSC030-11|NRS030|BOLD:AAD2426
Channa micropeltes|DSCHA020-07|CHAN024|BOLD:AAD2426
Channa micropeltes|NRSC029-11|NRS029|BOLD:AAD2426
Channa micropeltes|DSCHA028-07|CHAN032|BOLD:AAD2426
Channa micropeltes|DSCHA017-07|CHAN021|BOLD:AAD2426
Channa pleurophthalmus|DSCHA014-07|CHAN018|BOLD:AAI7162
Channa pleurophthalmus|DSCHA015-07|CHAN019|BOLD:AAI7162
Channa punctata|DSCHA076-13|DAN02-166.10|BOLD:ACG5323
Channa punctata|DSCHA077-13|DAN09-166.11|BOLD:AAE8814
Channa punctata|DSCHA078-13|DAN09-163.13|BOLD:AAE8814
Channa punctata|ANGBF2432-12|JN245992|BOLD:AAE8814
Channa punctata|ANGBF2430-12|JN245990|BOLD:AAE8814
Channa punctata|ANGBF2415-12|HM117201|BOLD:AAE8814
Channa punctata|ANGBF2413-12|HM117199|BOLD:AAE8814
Channa punctata|ANGBF2414-12|HM117200|BOLD:AAE8814
Channa punctata|ANGBF2412-12|HM117198|BOLD:AAE8814
Channa punctata|ANGBF2411-12|HM117197|BOLD:AAE8814
Channa punctata|GBGC4216-08|EU417795|BOLD:AAE8814
Channa punctata|ANGBF6001-12|FJ459410|BOLD:AAE8814
Channa punctata|GBGC4215-08|EU417796|BOLD:AAE8814
Channa punctata|GBGC4320-08|EU342202|BOLD:AAE8814
Channa punctata|GBGC4321-08|EU342201|BOLD:AAE8814
Channa punctata|ANGBF2442-12|FJ459409|BOLD:AAE8814
Channa punctata|ANGBF2441-12|FJ459408|BOLD:AAE8814
Channa melasoma|NRSC008-11|NRS-008|BOLD:ABW1864
Channa striata|GBGC4319-08|EU342203|BOLD:AAB2498
Channa striata|GBGC4318-08|EU342204|BOLD:AAB2498
Channa striata|ANGBF2429-12|JN245989|BOLD:ACB7973
Channa striata|ANGBF2416-12|HM117202|BOLD:ACB7973
Channa striata|ANGBF2418-12|HM117204|BOLD:ACB7973
Channa striata|ANGBF2419-12|HM117205|BOLD:ACB7973
Channa striata|ANGBF2420-12|HM117206|BOLD:ACB7973
Channa striata|ANGBF2417-12|HM117203|BOLD:ACB7973
Channa striata|NRSC011-11|NRS011|BOLD:AAB2497
Channa striata|PARO015-05|CHAN003|BOLD:AAB2497
Channa striata|GBGCA4818-13|KC819608|BOLD:AAB2497
Channa striata|ANGBF2448-12|JQ661364|BOLD:AAB2497
Channa striata|NRSC018-11|NRS018|BOLD:AAB2497
Channa striata|BLB007-10|Cstri3-LdB|BOLD:AAB2497
Channa striata|BLB005-10|Cstri1-LdB|BOLD:AAB2497
Channa striata|BTL035-10|Cstr4|BOLD:AAB2497
Channa striata|BTL033-10|Cstr2|BOLD:AAB2497
Channa striata|BTL032-10|Cstr1|BOLD:AAB2497
Channa striata|BTL034-10|Cstr3|BOLD:AAB2497
Channa striata|BTL036-10|Cstr5|BOLD:AAB2497
Channa striata|BLB006-10|Cstri2-LdB|BOLD:AAB2497
Channa striata|BLB008-10|Cstri4-LdB|BOLD:AAB2497
Channa striata|BLB009-10|Cstri5-LdB|BOLD:AAB2497
Channa striata|NRSC032-11|NRS032|BOLD:AAB2497

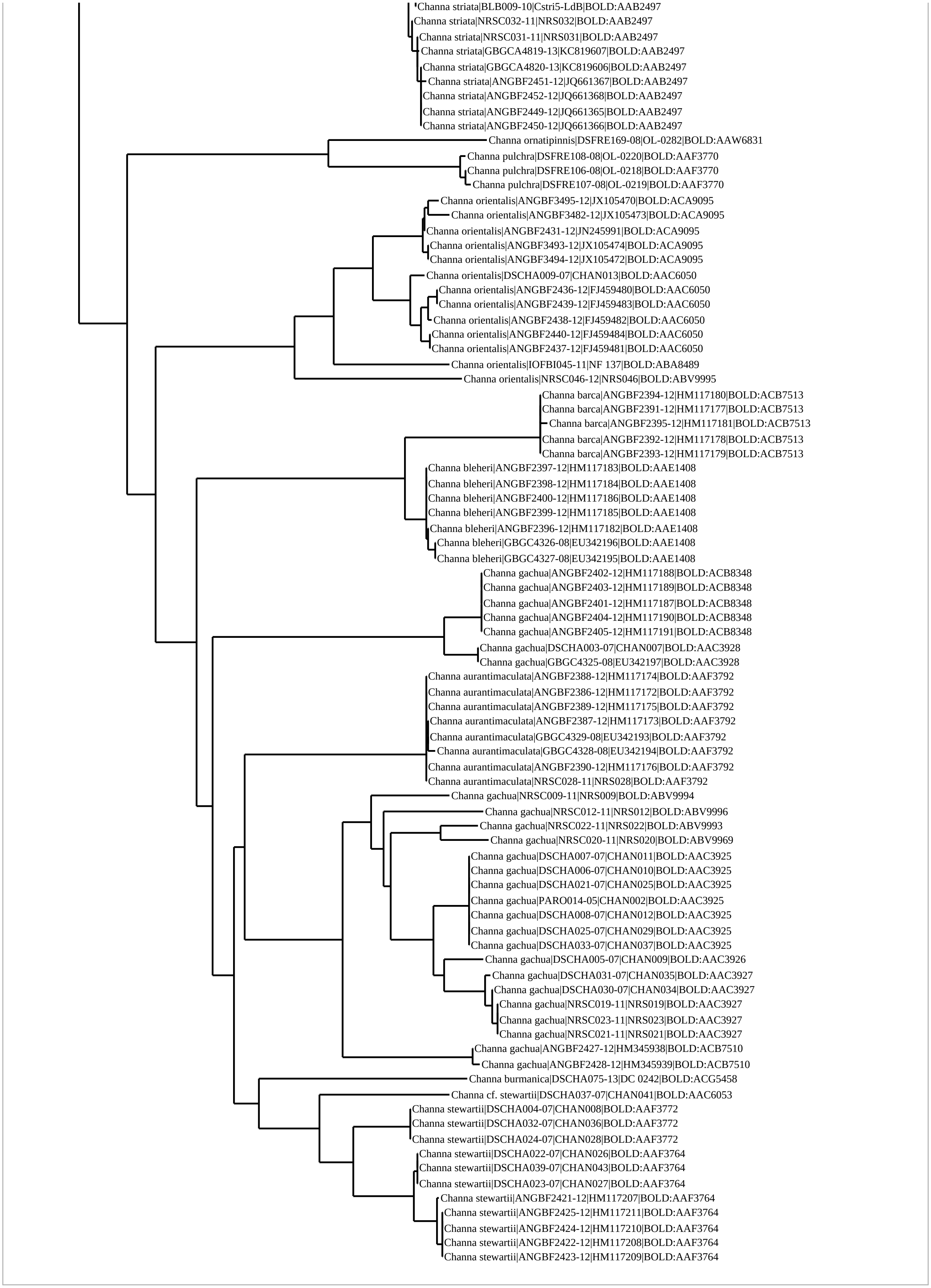
Channa striata|BLB009-10|Cstri5-LdB|BOLD:AAB2497
Channa striata|NRSC032-11|NRS032|BOLD:AAB2497
Channa striata|NRSC031-11|NRS031|BOLD:AAB2497
Channa striata|GBGCA4819-13|KC819607|BOLD:AAB2497
Channa striata|GBGCA4820-13|KC819606|BOLD:AAB2497
Channa striata|ANGBF2451-12|JQ661367|BOLD:AAB2497
Channa striata|ANGBF2452-12|JQ661368|BOLD:AAB2497
Channa striata|ANGBF2449-12|JQ661365|BOLD:AAB2497
Channa striata|ANGBF2450-12|JQ661366|BOLD:AAB2497
Channa ornatipinnis|DSFRE169-08|OL-0282|BOLD:AAW6831
Channa pulchra|DSFRE108-08|OL-0220|BOLD:AAF3770
Channa pulchra|DSFRE106-08|OL-0218|BOLD:AAF3770
Channa pulchra|DSFRE107-08|OL-0219|BOLD:AAF3770
Channa orientalis|ANGBF3495-12|JX105470|BOLD:ACA9095
Channa orientalis|ANGBF3482-12|JX105473|BOLD:ACA9095
Channa orientalis|ANGBF2431-12|JN245991|BOLD:ACA9095
Channa orientalis|ANGBF3493-12|JX105474|BOLD:ACA9095
Channa orientalis|ANGBF3494-12|JX105472|BOLD:ACA9095
Channa orientalis|DSCHA009-07|CHAN013|BOLD:AAC6050
Channa orientalis|ANGBF2436-12|FJ459480|BOLD:AAC6050
Channa orientalis|ANGBF2439-12|FJ459483|BOLD:AAC6050
Channa orientalis|ANGBF2438-12|FJ459482|BOLD:AAC6050
Channa orientalis|ANGBF2440-12|FJ459484|BOLD:AAC6050
Channa orientalis|ANGBF2437-12|FJ459481|BOLD:AAC6050
Channa orientalis|IOFBI045-11|NF 137|BOLD:ABA8489
Channa orientalis|NRSC046-12|NRS046|BOLD:ABV9995
Channa barca|ANGBF2394-12|HM117180|BOLD:ACB7513
Channa barca|ANGBF2391-12|HM117177|BOLD:ACB7513
Channa barca|ANGBF2395-12|HM117181|BOLD:ACB7513
Channa barca|ANGBF2392-12|HM117178|BOLD:ACB7513
Channa barca|ANGBF2393-12|HM117179|BOLD:ACB7513
Channa bleheri|ANGBF2397-12|HM117183|BOLD:AAE1408
Channa bleheri|ANGBF2398-12|HM117184|BOLD:AAE1408
Channa bleheri|ANGBF2400-12|HM117186|BOLD:AAE1408
Channa bleheri|ANGBF2399-12|HM117185|BOLD:AAE1408
Channa bleheri|ANGBF2396-12|HM117182|BOLD:AAE1408
Channa bleheri|GBGC4326-08|EU342196|BOLD:AAE1408
Channa bleheri|GBGC4327-08|EU342195|BOLD:AAE1408
Channa gachua|ANGBF2402-12|HM117188|BOLD:ACB8348
Channa gachua|ANGBF2403-12|HM117189|BOLD:ACB8348
Channa gachua|ANGBF2401-12|HM117187|BOLD:ACB8348
Channa gachua|ANGBF2404-12|HM117190|BOLD:ACB8348
Channa gachua|ANGBF2405-12|HM117191|BOLD:ACB8348
Channa gachua|DSCHA003-07|CHAN007|BOLD:AAC3928
Channa gachua|GBGC4325-08|EU342197|BOLD:AAC3928
Channa aurantimaculata|ANGBF2388-12|HM117174|BOLD:AAF3792
Channa aurantimaculata|ANGBF2386-12|HM117172|BOLD:AAF3792
Channa aurantimaculata|ANGBF2389-12|HM117175|BOLD:AAF3792
Channa aurantimaculata|ANGBF2387-12|HM117173|BOLD:AAF3792
Channa aurantimaculata|GBGC4329-08|EU342193|BOLD:AAF3792
Channa aurantimaculata|GBGC4328-08|EU342194|BOLD:AAF3792
Channa aurantimaculata|ANGBF2390-12|HM117176|BOLD:AAF3792
Channa aurantimaculata|NRSC028-11|NRS028|BOLD:AAF3792
Channa gachua|NRSC009-11|NRS009|BOLD:ABV9994
Channa gachua|NRSC012-11|NRS012|BOLD:ABV9996
Channa gachua|NRSC022-11|NRS022|BOLD:ABV9993
Channa gachua|NRSC020-11|NRS020|BOLD:ABV9969
Channa gachua|DSCHA007-07|CHAN011|BOLD:AAC3925
Channa gachua|DSCHA006-07|CHAN010|BOLD:AAC3925
Channa gachua|DSCHA021-07|CHAN025|BOLD:AAC3925
Channa gachua|PARO014-05|CHAN002|BOLD:AAC3925
Channa gachua|DSCHA008-07|CHAN012|BOLD:AAC3925
Channa gachua|DSCHA025-07|CHAN029|BOLD:AAC3925
Channa gachua|DSCHA033-07|CHAN037|BOLD:AAC3925
Channa gachua|DSCHA005-07|CHAN009|BOLD:AAC3926
Channa gachua|DSCHA031-07|CHAN035|BOLD:AAC3927
Channa gachua|DSCHA030-07|CHAN034|BOLD:AAC3927
Channa gachua|NRSC019-11|NRS019|BOLD:AAC3927
Channa gachua|NRSC023-11|NRS023|BOLD:AAC3927
Channa gachua|NRSC021-11|NRS021|BOLD:AAC3927
Channa gachua|ANGBF2427-12|HM345938|BOLD:ACB7510
Channa gachua|ANGBF2428-12|HM345939|BOLD:ACB7510
Channa burmanica|DSCHA075-13|DC 0242|BOLD:ACG5458
Channa cf. stewartii|DSCHA037-07|CHAN041|BOLD:AAC6053
Channa stewartii|DSCHA004-07|CHAN008|BOLD:AAF3772
Channa stewartii|DSCHA032-07|CHAN036|BOLD:AAF3772
Channa stewartii|DSCHA024-07|CHAN028|BOLD:AAF3772
Channa stewartii|DSCHA022-07|CHAN026|BOLD:AAF3764
Channa stewartii|DSCHA039-07|CHAN043|BOLD:AAF3764
Channa stewartii|DSCHA023-07|CHAN027|BOLD:AAF3764
Channa stewartii|ANGBF2421-12|HM117207|BOLD:AAF3764
Channa stewartii|ANGBF2425-12|HM117211|BOLD:AAF3764
Channa stewartii|ANGBF2424-12|HM117210|BOLD:AAF3764
Channa stewartii|ANGBF2422-12|HM117208|BOLD:AAF3764
Channa stewartii|ANGBF2423-12|HM117209|BOLD:AAF3764
