# Supplementary material for: Calibrating Snakehead Diversity with DNA Barcodes: Expanding Taxonomic Coverage to Enable Identification of Potential and Established Invasive Species
Source: PLoS One. 2014 Jun 10;9(6):e99546. doi: 10.1371/journal.pone.0099546 (PMC4051700; doi:10.1371/journal.pone.0099546)
Supplement: Table S1 — Table of species name, process ID, sample ID, country specimen was sourced from, museum ID if applicable, if the specimen is from a native or non-native range and BIN number. (DOC) [file pone.0099546.s002.doc]

| **Identification** | **Process ID** | **Sample ID /Genbank accession Number** | **Country** | **Study; Museum ID where applicable** | **Reference / or Non-native** | **BIN Number** |
| --- | --- | --- | --- | --- | --- | --- |
| *C. argus* | DSCHA040-12 | KJ937443 | United States | This study | Non-native | ABW0047 |
|  | NRSC024-11 | KJ937412 | United States | This study; NCSM 30519 | Non-native | ABW0047 |
|  | DSCHA067-12 | KJ937354 | United States | This study | Non-native | ABW0047 |
|  | DSCHA043-12 | KJ937343 | United States | This study | Non-native | ABW0047 |
|  | NRSC026-11 | KJ937364 | United States | This study; NCSM 30519 | Non-native | ABW0047 |
|  | DSCHA072-12 | KJ937422 | United States | This study | Non-native | ABW0047 |
|  | NRSC004-11 | KJ937411 | United States | This study | Non-native | ABW0047 |
|  | ANGBF2445-12 | JQ358717 | China | Genbank, Unpublished | Reference | ABW0047 |
|  | ANGBF2447-12 | JQ358719 | China | Genbank, Unpublished | Reference | ABW0047 |
|  | CYTC2469-12 | NC_015191 | China | Genbank, Wang and Yang 2011 | Reference | ABW0048 |
|  | NRSC057-12 | KJ937396 | United States | This study | Non-native | ABW0047 |
|  | NRSC025-11 | KJ937446 | United States | This study; NCSM 30519 | Non-native | ABW0047 |
|  | NRSC006-11 | KJ937372 | United States | This study | Non-native | ABW0047 |
|  | DSCHA068-12 | KJ937409 | United States | This study | Non-native | ABW0047 |
|  | ANGBF2444-12 | JQ358716 | China | Genbank, Unpublished | Reference | ABW0047 |
|  | DSCHA069-12 | KJ937379 | United States | This study | Non-native | ABW0047 |
|  | NRSC003-11 | NRS003 | United States | This study | Non-native | no barcode |
|  | DSCHA042-12 | NRS064 | United States | This study | Non-native | no barcode |
|  | DSCHA071-12 | KJ937427 | United States | This study | Non-native | ABW0047 |
|  | DSCHA066-12 | KJ937438 | United States | This study | Non-native | ABW0047 |
|  | NRSC050-12 | KJ937356 | United States | This study | Non-native | ABW0047 |
|  | DSCHA041-12 | KJ937382 | United States | This study | Non-native | ABW0047 |
|  | DSCHA070-12 | KJ937344 | United States | This study | Non-native | ABW0047 |
|  | ANGBF2446-12 | JQ358718 | China | Genbank, Unpublished | Reference | ABW0047 |
|  | NRSC005-11 | KJ937457 | United States | This study | Non-native | ABW0047 |
|  | ANGBF2443-12 | JQ358715 | China | Genbank, Unpublished | Reference | ABW0047 |
|  | [GBMIN41061-13](javascript:openRecord('sequence',3306720);) | JX978723 | China | Genbank; Zhu et al. 2012 | Reference | ABW0047 |
|  | [GBGCA4836-13](javascript:openRecord('sequence',3653417);) | KC819590 | China | Genbank Zhu et al. 2013 | Reference | ABW0047 |
|  | [GBGCA4835-13](javascript:openRecord('sequence',3653416);) | KC819591 | China | Genbank Zhu et al. 2013 | Reference | ABW0047 |
|  | [GBGCA4834-13](javascript:openRecord('sequence',3653415);) | KC819592 | China | Genbank Zhu et al. 2013 | Reference | ABW0047 |
|  | [GBGCA4833-13](javascript:openRecord('sequence',3653414);) | KC819593 | China | Genbank Zhu et al. 2013 | Reference | ABW0047 |
|  | [GBGCA4832-13](javascript:openRecord('sequence',3653413);) | KC819594 | China | Genbank Zhu et al. 2013 | Reference | ABW0047 |
|  | [GBGCA4831-13](javascript:openRecord('sequence',3653412);) | KC819595 | China | Genbank Zhu et al. 2013 | Reference | ABW0047 |
|  | [GBGCA4830-13](javascript:openRecord('sequence',3653411);) | KC819596 | China | Genbank Zhu et al. 2013 | Reference | ABW0047 |
|  | [GBGCA4829-13](javascript:openRecord('sequence',3653410);) | KC819597 | China | Genbank Zhu et al. 2013 | Reference | ABW0047 |
|  | [GBGCA4828-13](javascript:openRecord('sequence',3653409);) | KC819598 | China | Genbank Zhu et al. 2013 | Reference | ABW0047 |
|  | [GBGCA4827-13](javascript:openRecord('sequence',3653408);) | KC819599 | China | Genbank Zhu et al. 2013 | Reference | ABW0047 |
|  | [GBGCA4826-13](javascript:openRecord('sequence',3653407);) | KC819600 | China | Genbank Zhu et al. 2013 | Reference | ABW0047 |
| *C. asiatica* | DSCHA036-07 | KJ937340 | n/a | This study | Reference | AAW6834 |
|  | [GBGCA4824-13](javascript:openRecord('sequence',3653405);) | KC819602 | China | Genbank; Zhu et al. 2013 | Reference | [BOLD:ACH5881](javascript:openBinPage('BOLD:ACH5881');) |
|  | [GBGCA4823-13](javascript:openRecord('sequence',3653404);) | KC819603 | China | Genbank; Zhu et al. 2013 | Reference | [BOLD:ACH5881](javascript:openBinPage('BOLD:ACH5881');) |
|  | [GBGCA4822-13](javascript:openRecord('sequence',3653403);) | KC819604 | China | Genbank; Zhu et al. 2013 | Reference | [BOLD:ACH5881](javascript:openBinPage('BOLD:ACH5881');) |
|  | [GBGCA4821-13](javascript:openRecord('sequence',3653402);) | KC819605 | China | Genbank; Zhu et al. 2013 | Reference | [BOLD:ACH5880](javascript:openBinPage('BOLD:ACH5880');) |
| *C. aurantimaculata* | GBGC4329-08 | EU342193 | India | Genbank, Benzinger et al. 2011 | Reference | AAF3792 |
|  | ANGBF2389-12 | HM117175 | India | Genbank; Lakra et al. 2010 | Reference | AAF3792 |
|  | ANGBF2386-12 | HM117172 | India | Genbank; Lakra et al. 2010 | Reference | AAF3792 |
|  | NRSC028-11 | KJ937387 | United States | This study; NCSM 40915 | Non-native | AAF3792 |
|  | ANGBF2388-12 | HM117174 | India | Genbank; Lakra et al. 2010 | Reference | AAF3792 |
|  | GBGC4328-08 | EU342194 | India | Genbank, Benzinger et al. 2011 | Reference | AAF3792 |
|  | ANGBF2387-12 | HM117173 | India | Genbank; Lakra et al. 2010 | Reference | AAF3792 |
|  | ANGBF2390-12 | HM117176 | India | Genbank; Lakra et al. 2010 | Reference | AAF3792 |
| *C. bankanensis* | DSCHA035-07 | KJ937371 | n/a | This study | Reference | AAI7246 |
|  | DSCHA034-07 | KJ937395 | n/a | This study | Reference | AAI7246 |
| *C. barca* | ANGBF2395-12 | HM117181 | India | Genbank; Lakra et al. 2010 | Reference | ACB7513 |
|  | ANGBF2392-12 | HM117178 | India | Genbank; Lakra et al. 2010 | Reference | ACB7513 |
|  | ANGBF2394-12 | HM117180 | India | Genbank; Lakra et al. 2010 | Reference | ACB7513 |
|  | ANGBF2393-12 | HM117179 | India | Genbank; Lakra et al. 2010 | Reference | ACB7513 |
|  | ANGBF2391-12 | HM117177 | India | Genbank; Lakra et al. 2010 | Reference | ACB7513 |
| *C. bleheri* | ANGBF2400-12 | HM117186 | India | Genbank; Lakra et al. 2010 | Reference | AAE1408 |
|  | GBGC4327-08 | EU342195 | India | Genbank, Benzinger et al. 2011 | Reference | AAE1408 |
|  | ANGBF2397-12 | HM117183 | India | Genbank; Lakra et al. 2010 | Reference | AAE1408 |
|  | ANGBF2398-12 | HM117184 | India | Genbank; Lakra et al. 2010 | Reference | AAE1408 |
|  | ANGBF2399-12 | HM117185 | India | Genbank; Lakra et al. 2010 | Reference | AAE1408 |
|  | ANGBF2396-12 | HM117182 | India | Genbank; Lakra et al. 2010 | Reference | AAE1408 |
|  | GBGC4326-08 | EU342196 | India | Genbank, Benzinger et al. 2011 | Reference | AAE1408 |
| *C. burmanica* | [DSCHA075-13](javascript:openRecord('sequence',3411025);) | KJ937381 | Myanmar | This study; CAS 235520 | Reference | ACG5458 |
| *C. diplogramme* | DSCHA045-12 | CD5.1 | India | This study | Reference | no barcode |
|  | GBGC4312-08 | EU342210 | India | Genbank, Benzinger et al. 2011 | Reference | AAD7592 |
|  | NRSC040-11 | KJ937445 | India | This study; Adamson et al. 2010 | Reference | AAD7592 |
|  | GBGC4316-08 | EU342206 | India | Genbank, Benzinger et al. 2011 | Reference | AAD7592 |
|  | DSCHA047-12 | CM5 | India | This study | Reference | no barcode |
|  | NRSC045-12 | KJ937360 | India | This study | Reference | AAD7592 |
|  | GBGC4313-08 | EU342209 | India | Genbank, Benzinger et al. 2011 | Reference | AAD7592 |
|  | GBGC4315-08 | EU342207 | India | Genbank, Benzinger et al. 2011 | Reference | AAD7592 |
|  | GBGC4314-08 | EU342208 | India | Genbank, Benzinger et al. 2011 | Reference | AAD7592 |
|  | GBGC4317-08 | EU342205 | India | Genbank, Benzinger et al. 2011 | Reference | AAD7592 |
|  | DSCHA044-12 | CD4 | India | This study | Reference | no barcode |
|  | NRSC043-12 | KJ937431 | India | This study | Reference | AAD7592 |
|  | NRSC044-12 | KJ937448 | India | This study | Reference | AAD7592 |
| *C. gachua* | NRSC021-11 | KJ937423 | Thailand | This study; UF 176414 | Reference | [AAC3927](javascript:openBinPage('BOLD:AAC3927');) |
|  | DSCHA025-07 | KJ937363 | n/a | This study | Reference | [AAC3925](javascript:openBinPage('BOLD:AAC3925');) |
|  | DSCHA055-12 | CG4 | India | This study | Reference | no barcode |
|  | NRSC023-11 | KJ937456 | Thailand | This study; UF 176567 | Reference | [AAC3927](javascript:openBinPage('BOLD:AAC3927');) |
|  | ANGBF2405-12 | HM117191 | India | Genbank; Lakra et al. 2010 | Reference | [ACB8348](javascript:openBinPage('BOLD:ACB8348');) |
|  | DSCHA005-07 | KJ937367 | Myanmar | This study | Reference | [AAC3926](javascript:openBinPage('BOLD:AAC3926');) |
|  | NRSC019-11 | KJ937361 | Thailand | This study; UF 176379 | Reference | [AAC3927](javascript:openBinPage('BOLD:AAC3927');) |
|  | ANGBF2402-12 | HM117188 | India | Genbank; Lakra et al. 2010 | Reference | [ACB8348](javascript:openBinPage('BOLD:ACB8348');) |
|  | DSCHA003-07 | KJ937393 | India | This study | Reference | [AAC3928](javascript:openBinPage('BOLD:AAC3928');) |
|  | ANGBF2428-12 | HM345939 | Indonesia | Genbank; Unpublished | Reference | [ACB7510](javascript:openBinPage('BOLD:ACB7510');) |
|  | NRSC022-11 | KJ937342 | Thailand | This study; UF 176431 | Reference | [ABV9993](javascript:openBinPage('BOLD:ABV9993');) |
|  | GBGC4325-08 | EU342197 | India | Genbank, Benzinger et al. 2011 | Reference | [AAC3928](javascript:openBinPage('BOLD:AAC3928');) |
|  | ANGBF2403-12 | HM117189 | India | Genbank; Lakra et al. 2010 | Reference | [ACB8348](javascript:openBinPage('BOLD:ACB8348');) |
|  | DSCHA007-07 | KJ937339 | Myanmar | This study | Reference | [AAC3925](javascript:openBinPage('BOLD:AAC3925');) |
|  | PARO014-05 | KJ937404 | Thailand | This study | Reference | [AAC3925](javascript:openBinPage('BOLD:AAC3925');) |
|  | ANGBF2401-12 | HM117187 | India | Genbank; Lakra et al. 2010 | Reference | [ACB8348](javascript:openBinPage('BOLD:ACB8348');) |
|  | DSCHA006-07 | KJ937365 | Myanmar | This study | Reference | [AAC3925](javascript:openBinPage('BOLD:AAC3925');) |
|  | DSCHA008-07 | KJ937435 | Myanmar | This study | Reference | [AAC3925](javascript:openBinPage('BOLD:AAC3925');) |
|  | ANGBF2427-12 | HM345938 | Indonesia | Genbank; Unpublished | Reference | [ACB7510](javascript:openBinPage('BOLD:ACB7510');) |
|  | NRSC020-11 | KJ937370 | Thailand | This study; UF 176398 | Reference | [ABV9969](javascript:openBinPage('BOLD:ABV9969');) |
|  | DSCHA033-07 | KJ937359 | Thailand | This study | Reference | [AAC3925](javascript:openBinPage('BOLD:AAC3925');) |
|  | NRSC009-11 | KJ937429 | Thailand | This study (UF voucher) | Reference | [ABV9994](javascript:openBinPage('BOLD:ABV9994');) |
|  | DSCHA031-07 | KJ937368 | Thailand | This study | Reference | [AAC3927](javascript:openBinPage('BOLD:AAC3927');) |
|  | DSCHA030-07 | KJ937353 | Thailand | This study | Reference | [AAC3927](javascript:openBinPage('BOLD:AAC3927');) |
|  | DSCHA053-12 | CG2 | India | This study | Reference | no barcode |
|  | DSCHA021-07 | KJ937366 | n/a | This study | Reference | [AAC3925](javascript:openBinPage('BOLD:AAC3925');) |
|  | DSCHA056-12 | CG5.1 | India | This study | Reference | no barcode |
|  | ANGBF2404-12 | HM117190 | India | Genbank; Lakra et al. 2010 | Reference | [ACB8348](javascript:openBinPage('BOLD:ACB8348');) |
|  | DSCHA054-12 | CG3 | India | This study | Reference | no barcode |
|  | NRSC015-11 | NRS015 | Thailand | This study; UF 173359 | Reference | no barcode |
|  | NRSC012-11 | KJ937358 | Thailand | This study; UF 169929 | Reference | [ABV9996](javascript:openBinPage('BOLD:ABV9996');) |
| *C. lucius* | NRSC013-11 | KJ937397 | Thailand | This study; UF 173354 | Reference | ABW0051 |
|  | NRSC060-12 | KJ937430 | Malaysia | This study | Reference | [ABW0051](javascript:openBinPage('BOLD:ABW0051');) |
|  | NRSC016-11 | NRS016 | Thailand | This study; UF 173360 | Reference | no barcode |
|  | NRSC059-12 | KJ937399 | Malaysia | This study | Reference | [ABW0051](javascript:openBinPage('BOLD:ABW0051');) |
|  | NRSC017-11 | KJ937433 | Thailand | This study; UF 173363 | Reference | [ABW0051](javascript:openBinPage('BOLD:ABW0051');) |
|  | NRSC058-12 | KJ937385 | Malaysia | This study | Reference | [AAW6833](javascript:openBinPage('BOLD:AAW6833');) |
|  | NRSC061-12 | KJ937389 | Malaysia | This study | Reference | [ABW0051](javascript:openBinPage('BOLD:ABW0051');) |
| *C. maculata* | NRSC027-11 | KJ937400 | United States | This study; NCSM 53258 | Non-native | [ABW0048](javascript:openBinPage('BOLD:ABW0048');) |
|  | NRSC007-11 | KJ937386 | Canada | This study | Non-native | [ABW0048](javascript:openBinPage('BOLD:ABW0048');) |
|  | NRSC042-11 | KJ937459 | Vietnam | This study | Reference | [ABW0048](javascript:openBinPage('BOLD:ABW0048');) |
|  | CYTC5828-13 | KC310861 | China | Genbank; Wang et al. 2013 | Reference | [ABW0048](javascript:openBinPage('BOLD:ABW0048');) |
|  | [GBMIN41062-13](javascript:openRecord('sequence',3306721);) | JX978724 | China | Zhu et al. 2012 | Reference | [ABW0048](javascript:openBinPage('BOLD:ABW0048');) |
|  | GBGCA4825-13 | KC819601 | China | Genbank Zhu et al. 2013 | Reference | [ABW0048](javascript:openBinPage('BOLD:ABW0048');) |
| *C. marulius* | NRSC048-12 | KJ937341 | India | This study | Reference | [AAI7187](javascript:openBinPage('BOLD:AAI7187');) |
|  | NRSC049-12 | KJ937388 | India | This study | Reference | [AAI7187](javascript:openBinPage('BOLD:AAI7187');) |
|  | NRSC052-12 | KJ937432 | United States | This study; UF 5052 | Non-native | [ABW0012](javascript:openBinPage('BOLD:ABW0012');) |
|  | NRSC053-12 | KJ937352 | United States | This study; UF 5053 | Non-native | [ABW0012](javascript:openBinPage('BOLD:ABW0012');) |
|  | NRSC055-12 | KJ937407 | United States | This study; UF 5055 | Non-native | [ABW0012](javascript:openBinPage('BOLD:ABW0012');) |
|  | NRSC056-12 | KJ937410 | United States | This study; UF 5056 | Non-native | [ABW0012](javascript:openBinPage('BOLD:ABW0012');) |
|  | NRSC047-12 | KJ937348 | India | This study | Reference | AAI7187 |
|  | NRSC051-12 | KJ937440 | United States | This study; UF 5051 | Non-native | ABW0012 |
|  | DSCHA046-12 | CM4 | India | This study | Reference | no barcode |
|  | NRSC001-11 | KJ937369 | Canada | This study; T00008 | Non-native | ABW0012 |
|  | NRSC054-12 | KJ937419 | United States | This study; UF5054 | Non-native | ABW0012 |
|  | ANGBF2410-12 | HM117196 | India | Genbank; Lakra et al. 2010 | Reference | AAI7187 |
|  | ANGBF2407-12 | HM117193 | India | Genbank; Lakra et al. 2010 | Reference | AAI7187 |
|  | ANGBF2406-12 | HM117192 | India | Genbank; Lakra et al. 2010 | Reference | AAI7187 |
|  | GBGC4322-08 | EU342200 | India | Genbank, Benzinger et al. 2011 | Reference | AAI7187 |
|  | GBGC4323-08 | EU342199 | India | Genbank, Benzinger et al. 2011 | Reference | AAI7187 |
|  | ANGBF2409-12 | HM117195 | India | Genbank; Lakra et al. 2010 | Reference | AAI7187 |
|  | ANGBF2408-12 | HM117194 | India | Genbank; Lakra et al. 2010 | Reference | AAI7187 |
| *C. melasoma* | NRSC008-11 | KJ937380 | Singapore | This study; (UF voucher) | Reference | [ABW1864](javascript:openBinPage('BOLD:ABW1864');) |
| *C. micropeltes* | NRSC029-11 | KJ937401 | United States | This study; NCSM 34668 | Non-native | [AAD2426](javascript:openBinPage('BOLD:AAD2426');) |
|  | NRSC030-11 | KJ937408 | United States | This study; NCSM 34668 | Non-native | [AAD2426](javascript:openBinPage('BOLD:AAD2426');) |
|  | DSCHA019-07 | KJ937402 | Thailand | This study | Reference | [AAD2426](javascript:openBinPage('BOLD:AAD2426');) |
|  | DSCHA027-07 | KJ937383 | Thailand | This study | Reference | [AAD2426](javascript:openBinPage('BOLD:AAD2426');) |
|  | DSCHA016-07 | KJ937394 | Thailand | This study | Reference | [AAD2426](javascript:openBinPage('BOLD:AAD2426');) |
|  | BNAF302-09 | CHMI-Petshop-1 | Canada | Genbank; April et al. 2011 | Non-native | [AAD2426](javascript:openBinPage('BOLD:AAD2426');) |
|  | NRSC002-11 | KJ937362 | Canada | This study; T00009 | Non-native | [AAD2426](javascript:openBinPage('BOLD:AAD2426');) |
|  | DSCHA028-07 | KJ937437 | Thailand | This study | Reference | [AAD2426](javascript:openBinPage('BOLD:AAD2426');) |
|  | BNAF303-09 | CHMI-Petshop-2 | Canada | Genbank; April et al. 2011 | Non-native | [AAD2426](javascript:openBinPage('BOLD:AAD2426');) |
|  | DSCHA020-07 | KJ937426 | Thailand | This study | Reference | [AAD2426](javascript:openBinPage('BOLD:AAD2426');) |
|  | DSCHA017-07 | KJ937375 | Thailand | This study | Non-native | [AAD2426](javascript:openBinPage('BOLD:AAD2426');) |
|  | DSCHA074-13 | KJ937458 | Canada | This study | Non-native | [AAD2426](javascript:openBinPage('BOLD:AAD2426');) |
| *C. orientalis* | IOFBI045-11 | NF 137 | India | Genbank; unpublished | Reference | [ABA8489](javascript:openBinPage('BOLD:ABA8489');) |
|  | DSCHA009-07 | KJ937436 | Myanmar | This study | Reference | [AAC6050](javascript:openBinPage('BOLD:AAC6050');) |
|  | ANGBF2436-12 | FJ459480 | India | Genbank; unpublished | Reference | [AAC6050](javascript:openBinPage('BOLD:AAC6050');) |
|  | ANGBF2438-12 | FJ459482 | India | Genbank; unpublished | Reference | [AAC6050](javascript:openBinPage('BOLD:AAC6050');) |
|  | ANGBF3493-12 | JX105474 | India | Genbank; unpublished | Reference | [ACA9095](javascript:openBinPage('BOLD:ACA9095');) |
|  | ANGBF3482-12 | JX105473 | India | Genbank; unpublished | Reference | [ACA9095](javascript:openBinPage('BOLD:ACA9095');) |
|  | ANGBF3495-12 | JX105470 | India | Genbank; unpublished | Reference | [ACA9095](javascript:openBinPage('BOLD:ACA9095');) |
|  | ANGBF2439-12 | FJ459483 | India | Genbank; unpublished | Reference | AAC6050 |
|  | ANGBF2440-12 | FJ459484 | India | Genbank; unpublished | Reference | AAC6050 |
|  | NRSC046-12 | KJ937374 | India | This study | Reference | [ABV9995](javascript:openBinPage('BOLD:ABV9995');) |
|  | ANGBF2437-12 | FJ459481 | India | Genbank; unpublished | Reference | [AAC6050](javascript:openBinPage('BOLD:AAC6050');) |
|  | ANGBF2431-12 | JN245991 | India | Genbank; unpublished | Reference | [ACA9095](javascript:openBinPage('BOLD:ACA9095');) |
|  | ANGBF3494-12 | JX105472 | India | Genbank; unpublished | Reference | [ACA9095](javascript:openBinPage('BOLD:ACA9095');) |
| *C. ornatipinnis* | DSFRE169-08 | KJ937428 | n/a | This study | Reference | [AAW6831](javascript:openBinPage('BOLD:AAW6831');) |
| *C. panaw* | NRSC041-11 | KJ937403 | Denmark | This study; ZMUC P70523 | Non-native | [ABW1866](javascript:openBinPage('BOLD:ABW1866');) |
| *C. pleurophthalmus* | DSCHA014-07 | KJ937390 | n/a | This study | Reference | [AAI7162](javascript:openBinPage('BOLD:AAI7162');) |
|  | DSCHA015-07 | KJ937345 | n/a | This study | Reference | [AAI7162](javascript:openBinPage('BOLD:AAI7162');) |
| *C. pulchra* | DSFRE108-08 | KJ937349 | n/a | This study | Reference | [AAF3770](javascript:openBinPage('BOLD:AAF3770');) |
|  | DSFRE106-08 | KJ937434 | n/a | This study | Reference | [AAF3770](javascript:openBinPage('BOLD:AAF3770');) |
|  | DSFRE107-08 | KJ937442 | n/a | This study | Reference | [AAF3770](javascript:openBinPage('BOLD:AAF3770');) |
| *C. punctata* | ANGBF2415-12 | HM117201 | India | Genbank; Lakra et al. 2010 | Reference | [AAE8814](javascript:openBinPage('BOLD:AAE8814');) |
|  | GBGC4216-08 | EU417795 | India | Genbank, Benzinger et al. 2011 | Reference | [AAE8814](javascript:openBinPage('BOLD:AAE8814');) |
|  | ANGBF2411-12 | HM117197 | India | Genbank; Lakra et al. 2010 | Reference | [AAE8814](javascript:openBinPage('BOLD:AAE8814');) |
|  | ANGBF2442-12 | FJ459409 | India | Genbank; unpublished | Reference | [AAE8814](javascript:openBinPage('BOLD:AAE8814');) |
|  | ANGBF2432-12 | JN245992 | India | Genbank; unpublished | Reference | [AAE8814](javascript:openBinPage('BOLD:AAE8814');) |
|  | ANGBF2430-12 | JN245990 | India | Genbank; unpublished | Reference | [AAE8814](javascript:openBinPage('BOLD:AAE8814');) |
|  | GBGC4215-08 | EU417796 | India | Genbank, Benzinger et al. 2011 | Reference | [AAE8814](javascript:openBinPage('BOLD:AAE8814');) |
|  | ANGBF2441-12 | FJ459408 | India | Genbank; unpublished | Reference | [AAE8814](javascript:openBinPage('BOLD:AAE8814');) |
|  | ANGBF6001-12 | FJ459410 | India | Genbank; unpublished | Reference | [AAE8814](javascript:openBinPage('BOLD:AAE8814');) |
|  | GBGC4321-08 | EU342201 | India | Genbank, Benzinger et al. 2011 | Reference | [AAE8814](javascript:openBinPage('BOLD:AAE8814');) |
|  | GBGC4320-08 | EU342202 | India | Genbank, Benzinger et al. 2011 | Reference | [AAE8814](javascript:openBinPage('BOLD:AAE8814');) |
|  | ANGBF2413-12 | HM117199 | India | Genbank; Lakra et al. 2010 | Reference | [AAE8814](javascript:openBinPage('BOLD:AAE8814');) |
|  | ANGBF2414-12 | HM117200 | India | Genbank; Lakra et al. 2010 | Reference | [AAE8814](javascript:openBinPage('BOLD:AAE8814');) |
|  | ANGBF2412-12 | HM117198 | India | Genbank; Lakra et al. 2010 | Reference | [AAE8814](javascript:openBinPage('BOLD:AAE8814');) |
|  | [DSCHA077-13](javascript:openRecord('sequence',3411027);) | KJ937392 | Myanmar | This study; CAS 231313 | Reference | [AAE8814](javascript:openBinPage('BOLD:AAE8814');) |
|  | [DSCHA076-13](javascript:openRecord('sequence',3411026);) | KJ937377 | Myanmar | This study; CAS 231313 | Reference | [ACG5323](javascript:openBinPage('BOLD:AAE8814');) |
|  | [DSCHA078-13](javascript:openRecord('sequence',3411028);) | KJ937451 | Myanmar | This study; CAS 231292 | Reference | [AAE8814](javascript:openBinPage('BOLD:AAE8814');) |
| *C. sp.* | DSCHA059-12 | KJ937447 | Vietnam | This study | Non-native | ABW0048 |
|  | DSCHA065-12 | KJ937357 | Canada | This study | Non-native | ABW0048 |
|  | DSCHA060-12 | KJ937398 | Vietnam | This study | Non-native | ABW0048 |
|  | DSCHA061-12 | KJ937439 | Vietnam | This study | Non-native | ABW0048 |
|  | DSCHA064-12 | KJ937350 | Canada | This study | Non-native | ABW0048 |
|  | DSCHA057-12 | KJ937405 | Vietnam | This study | Non-native | ABW0048 |
|  | DSCHA063-12 | KJ937406 | Canada | This study | Non-native | ABW0048 |
|  | DSCHA062-12 | KJ937454 | Canada | This study | Non-native | ABW0048 |
|  | DSCHA058-12 | KJ937452 | Vietnam | This study | Non-native | ABW0048 |
|  | NRSC010-11 | KJ937355 | Indonesia | This study; UF 161557 | Reference | [ABW0050](javascript:openBinPage('BOLD:ABW0050');) |
| *C. cf. marulius* | [DSCHA002-07](javascript:openRecord('sequence',523520);) | KJ937378 | n/a | This study | Reference | [AAC6049](javascript:openBinPage('BOLD:AAC6049');) |
| *C. cf. stewartii* | [DSCHA037-07](javascript:openRecord('sequence',523555);) | KJ937384 | n/a | This study | Reference | [AAC6053](javascript:openBinPage('BOLD:AAC6053');) |
| *C. stewartii* | [DSCHA022-07](javascript:openRecord('sequence',523540);) | KJ937417 | India | This study | Reference | [AAF3764](http://www.boldsystems.org/index.php/Public_BarcodeCluster?clusterguid=BOLD:AAF3764) |
|  | DSCHA023-07 | KJ937347 | India | This study | Reference | AAF3764 |
|  | ANGBF2423-12 | HM117209 | India | Genbank; Lakra et al. 2010 | Reference | AAF3764 |
|  | DSCHA004-07 | KJ937416 | India | This study | Reference | [AAF3772](javascript:openBinPage('BOLD:AAF3772');) |
|  | ANGBF2425-12 | HM117211 | India | Genbank; Lakra et al. 2010 | Reference | AAF3764 |
|  | DSCHA032-07 | KJ937449 | Thailand | This study | Reference | AAF3772 |
|  | ANGBF2422-12 | HM117208 | India | Genbank; Lakra et al. 2010 | Reference | AAF3764 |
|  | DSCHA024-07 | KJ937420 | n/a | This study | Reference | AAF3772 |
|  | ANGBF2424-12 | HM117210 | India | Genbank; Lakra et al. 2010 | Reference | AAF3764 |
|  | [ANGBF2421-12](javascript:openRecord('sequence',2981825);) | [HM117207](javascript:openRecord('specimen',2981844);) | India | Genbank; Lakra et al. 2010 | Reference | AAF3764 |
|  | [DSCHA039-07](javascript:openRecord('sequence',523557);) | KJ937455 | India | This study | Reference | [AAF3764](http://www.boldsystems.org/index.php/Public_BarcodeCluster?clusterguid=BOLD:AAF3764) |
| *C. striata* | ANGBF2451-12 | JQ661367 | Thailand | Genbank; unpublished | Reference | AAB2497 |
|  | BTL036-10 | Cstr5 | Philippines | Genbank, Aquilino et al. 2011 | Reference | AAB2497 |
|  | BLB005-10 | Cstri1-LdB | Philippines | Genbank, Aquino et al. 2011 | Reference | AAB2497 |
|  | ANGBF2449-12 | JQ661365 | Thailand | Genbank; unpublished | Reference | AAB2497 |
|  | BLB006-10 | Cstri2-LdB | Philippines | Genbank, Aquino et al. 2011 | Reference | AAB2497 |
|  | ANGBF2416-12 | HM117202 | India | Genbank; Lakra et al. 2010 | Reference | ACB7973 |
|  | ANGBF2420-12 | HM117206 | India | Genbank; Lakra et al. 2010 | Reference | ACB7973 |
|  | BLB007-10 | Cstri3-LdB | Philippines | Genbank, Aquino et al. 2011 | Reference | AAB2497 |
|  | NRSC031-11 | KJ937373 | United States | This study; CU 91899 | Non-native | AAB2497 |
|  | ANGBF2448-12 | JQ661364 | Thailand | Genbank; unpublished | Reference | AAB2497 |
|  | NRSC014-11 | NRS014 | Thailand | This study; UF 173357 | Reference | AAB2497 |
|  | GBGC4318-08 | EU342204 | India | Genbank, Benzinger et al. 2011 | Reference | AAB2498 |
|  | DSCHA051-12 | CS4 | India | This study | Reference | no barcode |
|  | BLB008-10 | Cstri4-LdB | Philippines | Genbank, Aquino et al. 2011 | Reference | AAB2497 |
|  | BLB009-10 | Cstri5-LdB | Philippines | Genbank, Aquino et al. 2011 | Reference | AAB2497 |
|  | ANGBF2452-12 | JQ661368 | Thailand | Genbank; unpublished | Reference | AAB2497 |
|  | DSCHA052-12 | CS5 | India | This study | Reference | no barcode |
|  | ANGBF2429-12 | JN245989 | India | Genbank; unpublished | Reference | ACB7973 |
|  | DSCHA049-12 | CS2 | India | This study | Reference | no barcode |
|  | NRSC032-11 | KJ937450 | United States | This study; CU 91900 | Non-native | AAB2497 |
|  | NRSC018-11 | KJ937376 | Thailand | This study; UF 173364 | Reference | AAB2497 |
|  | ANGBF2419-12 | HM117205 | India | Genbank; Lakra et al. 2010 | Reference | ACB7973 |
|  | GBGC4319-08 | EU342203 | India | Genbank, Benzinger et al. 2011 | Reference | AAB2498 |
|  | DSCHA050-12 | CS3 | India | This study | Reference | no barcode |
|  | ANGBF2417-12 | HM117203 | India | Genbank; Lakra et al. 2010 | Reference | ACB7973 |
|  | ANGBF2450-12 | JQ661366 | Thailand | Genbank; unpublished | Reference | AAB2497 |
|  | ANGBF2418-12 | HM117204 | India | Genbank; Lakra et al. 2010 | Reference | ACB7973 |
|  | BTL032-10 | Cstr1 | Philippines | Genbank, Aquilino et al. 2011 | Reference | AAB2497 |
|  | BTL033-10 | Cstr2 | Philippines | Genbank, Aquilino et al. 2011 | Reference | AAB2497 |
|  | BTL034-10 | Cstr3 | Philippines | Genbank, Aquilino et al. 2011 | Reference | AAB2497 |
|  | BTL035-10 | Cstr4 | Philippines | Genbank, Aquilino et al. 2011 | Reference | AAB2497 |
|  | DSCHA048-12 | CS1 | India | This study | Reference | no barcode |
|  | PARO015-05 | KJ937425 | Indonesia | This study | Reference | AAB2497 |
|  | NRSC011-11 | KJ937421 | Indonesia | This study; UF 162793 | Reference | AAB2497 |
|  | [GBGCA4820-13](javascript:openRecord('sequence',3653401);) | KC819606 | China | Genbank Zhu et al. 2013 | Reference | [BOLD:AAB2497](javascript:openBinPage('BOLD:AAB2497');) |
|  | [GBGCA4819-13](javascript:openRecord('sequence',3653400);) | KC819607 | China | Genbank Zhu et al. 2013 | Reference | [BOLD:AAB2497](javascript:openBinPage('BOLD:AAB2497');) |
|  | [GBGCA4818-13](javascript:openRecord('sequence',3653399);) | KC819608 | China | Genbank Zhu et al. 2013 | Reference | [BOLD:AAB2497](javascript:openBinPage('BOLD:AAB2497');) |
| *P. sp.* | NRSC036-11 | NRS036 | Zambia | This study; CU 94229 | Reference | no barcode |
|  | NRSC037-11 | KJ937414 | Republic of the Congo | This study; CU 94850 | Reference | ABW0157 |
| *P. africana* | DSMIS258-11 | KJ937351 | Democratic Republic of the Congo | This study | Reference | AAF7843 |
|  | DSMIS256-11 | KJ937391 | Democratic Republic of the Congo | This study | Reference | AAF7843 |
|  | DSMIS257-11 | KJ937418 | Democratic Republic of the Congo | This study | Reference | AAF7843 |
| *P. insignis* | NRSC034-11 | KJ937441 | Republic of the Congo | This study; CU 92932 | Reference | ABW0157 |
|  | NRSC033-11 | KJ937413 | Republic of the Congo | This study; CU 92931 | Reference | ABW0157 |
|  | NRSC038-11 | KJ937415 | Democratic Republic of the Congo | This study; CU 96133 | Reference | ABW0157 |
|  | [NRSC035-11](javascript:openRecord('sequence',2165655);) | KJ937444 | Republic of the Congo | This study; CU 93144 | Reference | ABW0157 |
| *P. obscura* | BAFEN292-10 | BNF 292 | Nigeria | Genbank; Nwani et al. 2010 | Reference | AAF7842 |
|  | NRSC039-11 | KJ937453 | Democratic Republic of the Congo | This study; CU 96491 | Reference | AAF7843 |
|  | PARO016-05 | KJ937346 | Senegal | This study | Reference | AAF7842 |
|  | PARO017-05 | KJ937424 | Senegal | This study | Reference | AAF7842 |
|  | BAFEN284-10 | BNF 284 | Nigeria | Genbank; Nwani et al. 2010 | Reference | AAF7842 |
|  | BAFEN285-10 | BNF 285 | Nigeria | Genbank; Nwani et al. 2010 | Reference | AAF7842 |
|  | BAFEN291-10 | BNF 291 | Nigeria | Genbank; Nwani et al. 2010 | Reference | AAF7842 |
|  | [BAFEN290-10](javascript:openRecord('sequence',1485851);) | [BNF 290](javascript:openRecord('specimen',1485853);) | Nigeria | Genbank; Nwani et al. 2010 | Reference | AAF7842 |
| *C. argus (M)x C. maculata (F)* | [DSCHA073-12](javascript:openRecord('sequence',3041369);) | [JX978725](javascript:openRecord('specimen',3041388);) | China | Genbank; Zhu et al. 2012 | Reference | ABW0048 |
